# Accumulation of metabolic multimorbidity and its association with mortality: results from a prospective cohort study among people with HIV in China, 2010–2024

**DOI:** 10.1016/j.lanwpc.2026.101883

**Published:** 2026-05-22

**Authors:** Chenye Liu, Liqin Sun, Yun He, Fang Zhao, Xiaorui Li, Yinsong Luo, Dian Zhao, Hui Wu, Xi Xiao, Jinping Huang, Jinwei Wu, Yanjun Li, Hongzhou Lu, Ping Cen, Jiaye Liu

**Affiliations:** a School of Public Health, Shenzhen University Medical School, Shenzhen, Guangdong, China; b Department of Infectious Diseases, National Clinical Research Center for Infectious Diseases, Shenzhen Third People's Hospital, Shenzhen, Guangdong, China; c Infectious Disease Laboratory, Guangxi AIDS Clinical Treatment Center (Nanning), Nanning Fourth People's Hospital, Nanning, Guangxi, China

**Keywords:** HIV, PWH, Metabolic multimorbidity, Non-communicable diseases, Mortality

## Abstract

**Background:**

As survival among people with HIV (PWH) improves in the antiretroviral therapy (ART) era, metabolic comorbidities have become important determinants of long-term outcomes. However, longitudinal evidence quantifying the mortality impact of time-varying metabolic multimorbidity (MM) remains limited. This study aimed to characterise temporal trends in major metabolic conditions and MM patterns after ART initiation, and to quantify their associations with all-cause and non–AIDS-related mortality.

**Methods:**

We conducted a prospective two-centre cohort study of PWH who initiated ART in southern China between 2010 and 2024. Dyslipidaemia, diabetes, hypertension, metabolic dysfunction–associated steatotic liver disease, and osteoporosis were ascertained from clinical records and laboratory measurements, and MM was defined as the presence of at least two of these conditions. All-cause and non–AIDS-related deaths were identified through linkage with HIV surveillance, hospital records, and death registries. Associations between MM and mortality were evaluated using Cox regression models with MM treated as a time-varying exposure, and were reported as adjusted hazard ratios (aHRs) with 95% confidence intervals (CIs).

**Findings:**

Among 31,630 PWH who initiated ART between 2010 and 2024 (median age 35 years; 83·2% men), 3143 (9·9%) had MM at baseline, defined as present before or within 90 days after ART initiation. The incidence of all five metabolic conditions increased over time, with particularly marked rises after 2020. During follow-up, 8495 MM events occurred, most commonly as two-condition combinations of dyslipidaemia with diabetes or hypertension. All-cause mortality rates were 7·2 (95% CI 6·2–8·3), 10·3 (95% CI 9·7–10·8), and 15·9 (95% CI 14·5–17·4) per 1000 person-years among individuals with 0, 1, and ≥2 conditions, respectively. Compared with those with no metabolic condition, the aHRs for all-cause mortality were 1·7 (95% CI 1·5–2·0) for one condition and 2·1 (1·8–2·5) for ≥2 conditions; a similar dose–response relationship was observed for non–AIDS-related mortality. PWH with hypertension–diabetes–dyslipidaemia doublet or triad had substantially higher risks of all-cause mortality (aHR 2·1 and 4·1, respectively) and non–AIDS-related mortality (aHR 2·3 and 4·2, respectively) than those without metabolic comorbidity.

**Interpretation:**

As metabolic comorbidities accumulated over time, the burden of MM increased among PWH. Both overall metabolic burden and specific high–risk combinations were strongly associated with increased mortality. These findings underscore the need to move beyond virological control alone and to integrate structured metabolic comorbidity assessment and long-term metabolic risk management into routine HIV care in the ART era.

**Funding:**

National Natural Science Foundation of China, the Guangxi Key Research and Development Program, the project of the Guangdong Basic and Applied Basic Research Foundation, and the Guangdong Provincial Medical Science and Technology Research Fund Project.

**Research in context**

**Evidence before this study**

We searched PubMed and Web of Science for studies published from Jan 1, 2010, to December 31, 2025, using the search terms (HIV) AND (multimorbidity OR comorbidity) AND (metabolic OR dyslipidaemia OR diabetes OR hypertension OR osteoporosis OR “metabolic dysfunction–associated steatotic liver disease” OR MASLD). Existing evidence consistently indicates a growing burden of non-communicable diseases and multimorbidity among people with HIV (PWH) in the antiretroviral therapy (ART) era. Cardiometabolic conditions, particularly dyslipidaemia, hypertension and diabetes, are frequently reported and often cluster as PWH age and remain on long-term ART. Emerging cohort and mortality studies suggest that cardiometabolic causes now account for an increasing proportion of deaths among PWH. However, most available evidence is derived from cross-sectional analyses or baseline comorbidity assessments, with limited longitudinal data on the accumulation of metabolic conditions after ART initiation, the evolution of specific metabolic multimorbidity (MM) patterns over time, and their associations with mortality.

**Added value of this study**

In a large prospective two-centre cohort study in China, we characterised long-term trends in major metabolic comorbidities after ART initiation and examined their associations with mortality. By modelling both the number of metabolic conditions and specific combinations of conditions as time-varying exposures, we captured the accumulation of MM during routine HIV care and quantified its relationship with subsequent mortality. This approach demonstrated a consistent dose–response association between metabolic comorbidity burden and both all-cause and non–AIDS-related mortality. We further identified the most prevalent and clinically representative MM patterns, highlighting cardiometabolic combinations associated with particularly high mortality risk and providing a pragmatic framework for multimorbidity-based risk stratification in HIV services.

**Implications of all the available evidence**

As ART has transformed HIV into a chronic, manageable condition, non–AIDS-related comorbidities have become increasingly important determinants of long-term survival among PWH, with metabolic conditions accounting for a substantial share of this burden. The accumulating evidence indicates that metabolic comorbidity is now common in treated PWH and is associated with excess mortality, particularly among individuals with a high burden of metabolic disease and specific high–risk combinations. Sustaining further gains in life expectancy will therefore require a shift towards integrated HIV care models that prioritise early identification and longitudinal management of metabolic risk, supported by routine assessment of multimorbidity and closer coordination across cardiometabolic, hepatic, and bone health services.

## Introduction

With the widespread scale-up of antiretroviral therapy (ART), survival among people with HIV (PWH) has improved substantially.^1^, ^2^, ^3^ As AIDS-related illnesses have become better controlled, long-term health needs have shifted, and non-communicable chronic diseases (NCDs) now represent an increasing component of the overall disease burden in this population.^4^^,^^5^ These changes have placed greater emphasis on understanding chronic disease risk and management in the contemporary ART era.

Metabolic disorders account for a large proportion of the emerging NCD burden in PWH and often develop earlier and accumulate more rapidly than those in the general population.^3^ Dyslipidaemia, diabetes, and hypertension remain common metabolic comorbidities, while metabolic dysfunction–associated steatotic liver disease (MASLD) and bone disease are increasingly recognised as PWH age.^6^, ^7^, ^8^ Consistent with these shifts, non–AIDS-related causes now contribute more to deaths among PWH than AIDS-related conditions,^9^^,^^10^ highlighting the clinical importance of understanding metabolic health trajectories.

Despite increasing attention to NCDs in HIV care, longitudinal evidence on the development of metabolic comorbidities and metabolic multimorbidity (MM) remains scarce.^11^^,^^12^ In contemporary HIV research, multimorbidity has commonly been defined as the coexistence of chronic non-AIDS conditions, including hypertension, diabetes, dyslipidaemia, cardiovascular disease (CVD), chronic kidney disease (CKD), liver disease, malignancy, and, in some studies, common mental health disorders.^13^^,^^14^ Prior studies have generally shown that a greater comorbidity burden is associated with higher risks of mortality and cardiovascular events, although the magnitude of these associations varies across cohorts and endpoints.^15^^,^^16^ However, much of the existing literature has relied on comorbidity measures assessed at baseline or updated only intermittently, rather than modelling comorbidity status as a fully time-varying exposure throughout follow-up.^13^^,^^15^ Consequently, the extent to which the dynamic accumulation of metabolic comorbidities during long-term ART contributes to mortality among PWH remains insufficiently defined.

In this two-centre cohort of PWH initiating ART in southern China, we aimed to characterise temporal trends in major metabolic conditions and MM patterns after ART initiation, and to quantify the associations of time-dependent MM burden and specific MM combinations with all-cause and non–AIDS-related mortality.

## Methods

### Study design and population

We conducted a prospective two-centre cohort study of PWH who initiated ART between 1 January 2010 and 31 December 2024 at Shenzhen Third People's Hospital and Nanning Fourth People's Hospital, both of which are nationally designated HIV treatment centres in their respective cities in southern China, and were followed prospectively through routine clinical care. ART is provided free-of-charge in accordance with national HIV treatment guidelines. PWH attend routine follow-up visits approximately every three months for continuous ART prescriptions and health monitoring. Detailed baseline and follow-up assessments, including demographic characteristics, clinical diagnoses, and laboratory measurements, are collected as part of routine clinical care and recorded in the hospital's electronic medical record (EMR) systems. Further details on cohort characteristics, including prior studies using this dataset, are provided in the Supplementary Materials (Brief Introduction of the Cohort). As this was a prospective observational cohort study using data collected through routine clinical care and China's National HIV/AIDS Comprehensive Response Information Management System (CRIMS), written informed consent was obtained from participants at enrolment into HIV care in accordance with national regulations.

Eligible individuals were adults (≥18 years) with confirmed HIV infection who initiated ART during the study period and had at least 180 days of follow-up. We excluded participants with missing ART initiation dates, those lacking baseline demographic or clinical information in the EMR systems of Shenzhen or Nanning, and those with inconsistent or implausible data identified during quality control procedures. Follow-up began at ART initiation and continued until death or censoring at the earliest of the last clinical encounter or the end of the observation period (31 July 2025). After data cleaning and quality assurance procedures, 31,630 participants were included in the final analytical cohort.

### Data collection and definitions of metabolic conditions and MM

Participant records were obtained from the hospital EMR systems and CRIMS and underwent standardised cleaning and quality assurance procedures. Collected data included demographic characteristics (age at ART initiation, sex, and marital status); HIV-related variables (route of HIV transmission, categorised as heterosexual contact, men who have sex with men [MSM], injection drug use [IDU], and other routes, including mother-to-child transmission, blood transfusion, and occupational exposure; baseline CD4+ T-cell count; baseline HIV RNA viral load; initial ART regimen; and hepatitis B virus [HBV] or hepatitis C virus [HCV]); and laboratory indicators (white blood cell [WBC] count, haemoglobin [HB], platelet count, creatinine, fasting plasma glucose [FPG], alanine aminotransferase [ALT], aspartate aminotransferase [AST], high-density lipoprotein cholesterol [HDL-C], low-density lipoprotein cholesterol [LDL-C], total cholesterol [TC], and triglycerides [TG]. Initial ART regimens were categorised according to the class of the third agent combined with two nucleoside reverse transcriptase inhibitors (NRTIs) as integrase strand transfer inhibitor (INSTI)-based, non-nucleoside reverse transcriptase inhibitor (NNRTI)-based (efavirenz or nevirapine), protease inhibitor-based (ritonavir-boosted lopinavir), or other regimens.

The metabolic conditions of interest were dyslipidaemia, diabetes, hypertension, MASLD, and osteoporosis. These conditions were selected to represent major upstream metabolic domains and to capture primary metabolic dysfunction rather than established end-organ disease. Conditions such as CVD and CKD were therefore not included as components of MM because they reflect downstream organ damage that may arise from long-standing metabolic disturbance. MASLD and osteoporosis were included because they are increasingly recognised in ageing PWH and are relevant to long-term metabolic health.

These conditions were identified from routine administrative and clinical data using ICD-10 diagnosis codes and clinical-recorded diagnoses in EMR (Supplementary Table S1), supplemented when available by laboratory measurements according to guideline-recommended thresholds (e.g., lipid profile for dyslipidaemia and fasting plasma glucose for diabetes). In this study, these five conditions were considered metabolic comorbidities.^17^ MM was defined as the presence of at least two of these five conditions, consistent with the conventional definition of multimorbidity.^13^^,^^18^ This threshold was chosen to capture clinically relevant clustering of metabolically related conditions more broadly, whereas stricter thresholds (e.g., ≥3 conditions) may identify a narrower subgroup with greater disease burden and yield lower prevalence estimates. Baseline was defined as the date of ART initiation. For each participant, longitudinal diagnostic timelines were constructed to determine the onset of each metabolic condition and to track the accumulation and clustering of MM over time.

### Outcomes

The primary outcomes were all-cause mortality and non–AIDS-related mortality. In addition, we examined a composite metabolic-related non–AIDS-related mortality outcome, defined as non–AIDS deaths attributable to cardiovascular and cerebrovascular diseases and endocrine, nutritional and metabolic diseases. Cause-of-death information was obtained from both the hospital EMR and CRIMS. Deaths occurring in hospital were captured in the EMR, whereas out-of-hospital deaths were ascertained through routine follow-up in CRIMS and reconciled with available clinical records where possible.

To reduce outcome misclassification, two experienced HIV clinicians independently reviewed the recorded ICD-10 codes for death and applied prespecified criteria based on the WHO clinical staging system and the 1993 US Centers for Disease Control and Prevention AIDS case definition to confirm AIDS-related deaths.^19^ Any discrepancies were adjudicated by a third senior physician through consensus.

### Statistical analysis

Baseline characteristics were summarised descriptively, with categorical variables presented as counts and percentages as well as continuous variables as medians with interquartile ranges (IQRs). Differences between participants with and without baseline MM were compared using the chi-square test for categorical variables and the Wilcoxon rank-sum test for continuous variables, as appropriate.

Annual incidence rates of the five metabolic conditions were estimated by calendar year using person-time at risk. The prevalence of each metabolic condition was summarised at baseline, during follow-up, and at the end of observation for display in a radar plot. In addition, an UpSet plot was used to visualise the patterns of individual and co-occurring metabolic conditions at the end of follow-up. To further describe MM patterns and assess whether data–driven profiles were consistent with the co-occurrence patterns observed in the descriptive analyses, we performed an exploratory clustering analysis using partitioning around medoids (PAM) with Gower distance at prespecified timepoints of 1, 3, and 5 years after ART initiation, based on binary indicators of the five metabolic conditions.

To examine the association between MM accumulation and mortality, we fitted Cox regression models with MM modelled as a time-dependent exposure and estimated adjusted hazard ratios (aHRs) with 95% confidence intervals (CIs). Time since ART initiation was used as the underlying time scale to align the risk process with the post-ART accumulation of metabolic conditions. Analyses were landmarked at 90 days after ART initiation to allow a minimum period for baseline comorbidity ascertainment, to align with the routine 3-month follow-up schedule, and to reduce the influence of early events that might reflect pre-existing illness or acute clinical instability rather than subsequent accumulation of metabolic conditions. Any predefined metabolic condition documented before ART initiation or identified within 90 days after ART initiation was treated as a baseline condition. Missing covariate data (<5% for all variables) were handled using multiple imputation by chained equations.

Metabolic comorbidity was parameterised in two ways. One was based on the number of metabolic conditions (0, 1, and ≥2). The other used mutually exclusive combinations categories: no metabolic condition, any single condition, a hypertension–diabetes–dyslipidaemia doublet (defined as any two of these three conditions), a hypertension–diabetes–dyslipidaemia triad (defined as the coexistence of all three conditions), and a residual category including all remaining MM patterns involving MASLD and/or osteoporosis, with or without hypertension, diabetes, and dyslipidaemia. We used the categorisation of 0, 1, and ≥2 metabolic conditions as the primary parameterisation because it aligns with the operational definition of multimorbidity (≥2 conditions) while preserving adequate sample sizes and stable estimation across strata. The combination-based parameterisation was prespecified to enhance clinical interpretability by distinguishing patterns centred on hypertension, diabetes, and dyslipidaemia, which represent common upstream cardiometabolic risk factors. Less frequent patterns involving MASLD and/or osteoporosis were grouped as “other” to avoid sparse strata and unstable estimates. All categories were mutually exclusive to avoid double-counting of person-time.

To reduce simultaneity and potential reverse causality in the MM–mortality analyses, MM onset was defined as the diagnosis date of the second metabolic comorbidity, and deaths occurring within 90 days after MM onset were not attributed to MM exposure; this prespecified 90-day lag period was applied to reduce the likelihood that MM was ascertained during the terminal phase, in which MM onset and death might be recorded in close temporal proximity and thereby inflate associations due to reverse causation.

Models were adjusted for age at ART initiation, sex, marital status, route of HIV transmission, baseline CD4+ T-cell count, baseline HIV RNA viral load, initial ART regimen, HBV/HCV co-infection status, and routine laboratory indicators (e.g., WBC, platelet count, creatinine, FPG, ALT, AST, HDL-C, LDL-C, TC, and TG). These laboratory variables were included as covariates primarily to capture baseline physiological status, including systemic inflammatory burden, haematological condition, and hepatic and renal function, thereby reducing residual confounding related to overall health status at ART initiation. Sandwich variance estimators were used to account for within-person correlation arising from time-varying exposures. The proportional hazards assumption was evaluated using Schoenfeld residual tests and visual inspection of scaled Schoenfeld residual plots. Because evidence of non-proportional hazards was observed both globally and for the primary MM exposure variables, the overall Cox model estimates were interpreted as average associations over follow-up rather than as constant effects over time. To further characterise these time-varying associations, we performed piecewise Cox analyses using follow-up cutpoints informed by the proportional hazards assessment.^20^, ^21^, ^22^

Sensitivity analyses included a complete-case analysis using the observed data without multiple imputation and repetition of the primary mortality analyses using an alternative 180-day lag period for attributing MM to mortality. Additional analyses examined the associations of baseline MM and time-dependent MM with metabolic-related mortality. We also evaluated incident CVD and CKD as non-fatal outcomes related to metabolic burden using competing-risk regression with all-cause mortality treated as the competing event, and reported adjusted subdistribution hazard ratios (sHRs) with 95% CIs. The same exposure parameterisations and covariate adjustment strategy as in the primary analyses were applied.

All analyses were performed using R (version 4.4.1) and RStudio (version 2024.12.0). All tests were two-sided, and *p* values < 0·05 were considered statistically significant.

### Ethics approval

The study adhered to the ethical guidelines of the 1975 Declaration of Helsinki and was approved by the Institutional Review Board of Shenzhen University (PN-202500096).

### Role of the funding source

All funding entities were not involved in any aspect of the study, including research design, data collection, analysis or interpretation, manuscript drafting or revision, or decisions regarding publication. The corresponding author maintained authority over data stewardship and assumes sole responsibility for publication decisions.

## Results

### Baseline characteristics

Among 31,630 PWH included in the analysis, 26,307 (83·2%) were men and 5323 (16·8%) were women (Table 1). The median age at ART initiation was 35 years (IQR 28–48). At baseline, 3143 individuals (9·9%) had MM. PWH with baseline MM differed from those without baseline MM in age at ART initiation, marital status, HIV transmission route, and baseline CD4+ T-cell count category (all p < 0·001). Specifically, they were older at ART initiation, with a median age of 46 years (IQR 35–57) versus 34 years (IQR 28–46), and were more likely to be older than 45 years (50·2% vs 25·9%). They were also more often married (48·7% vs 38·7%) or divorced or widowed (17·4% vs 10·9%). Heterosexual transmission was more common among participants with baseline MM than among those without MM (56·5% vs 51·1%), whereas IDU was less frequent (0·8% vs 2·1%). A slightly higher proportion of participants with MM had a baseline CD4+ T-cell count of ≤200 cells/μL than those without baseline MM (41·2% vs 40·3%; *p* < 0·001).

**Table 1:** Baseline demographic and clinical characteristics by baseline MM status.

Characteristic	Overall N = 31,630	Baseline MM		*p* value
		No N = 28,487	Yes N = 3143	
Sex				0·075
Male	26,307 (83·2)	23,657 (83·0)	2650 (84·3)	
Female	5323 (16·8)	4830 (17·0)	493 (15·7)	
Age at ART initiation (years)				<0·001
18–25	4727 (14·9)	4588 (16·1)	139 (4·4)	
26–35	11,217 (35·5)	10,499 (36·9)	718 (22·8)	
36–45	6740 (21·3)	6032 (21·2)	708 (22·5)	
>45	8946 (28·3)	7368 (25·9)	1578 (50·2)	
Marital status				<0·001
Unmarried	15,302 (48·4)	14,247 (50·0)	1055 (33·6)	
Married	12,545 (39·7)	11,013 (38·7)	1532 (48·7)	
Divorced or widowed	3664 (11·6)	3116 (10·9)	548 (17·4)	
Others	119 (0·4)	111 (0·4)	8 (0·3)	
Route of HIV transmission				<0·001
IDU	627 (2·0)	602 (2·1)	25 (0·8)	
Heterosexual	16,336 (51·7)	14,560 (51·1)	1776 (56·5)	
MSM	14,413 (45·6)	13,087 (45·9)	1326 (42·2)	
Other	254 (0·8)	238 (0·8)	16 (0·5)	
Baseline CD4+ T-cell (cells/μL)				<0·001
≤200	12,770 (40·4)	11,474 (40·3)	1296 (41·2)	
201–350	10,645 (33·7)	9657 (33·9)	988 (31·4)	
351–500	5513 (17·4)	4993 (17·5)	520 (16·5)	
>500	2702 (8·5)	2363 (8·3)	339 (10·8)	
Baseline HIV RNA (copies/mL)				0·192
<5000	2108 (6·7)	1886 (6·6)	222 (7·1)	
5000–9999	1417 (4·5)	1294 (4·5)	123 (3·9)	
≥10,000	28,105 (88·9)	25,307 (88·8)	2798 (89·0)	
HBV co-infection				0·026
No	28,179 (89·1)	25,342 (89·0)	2837 (90·3)	
Yes	3451 (10·9)	3145 (11·0)	306 (9·7)	
HCV co-infection				<0·001
No	30,706 (97·1)	27,618 (97·0)	3088 (98·3)	
Yes	924 (2·9)	869 (3·1)	55 (1·8)	
ART regimen				<0·001
INSTI-based	4659 (14·7)	3795 (13·3)	864 (27·5)	
NNRTI-based	24,021 (75·9)	22,179 (77·9)	1842 (58·6)	
PI/r-based	2857 (9·0)	2438 (8·6)	419 (13·3)	
Others	93 (0·3)	75 (0·3)	18 (0·6)	
WBC (×10^9^/L)	5·3 (4·3–6·6)	5·3 (4·3–6·5)	5·5 (4·4–6·7)	<0·001
Platelet (×10^9^/L)	217·0 (176·0–262·0)	217·0 (176·0–262·0)	220·0 (175·0–266·0)	0·047
Creatinine (μmol/L)	73·0 (64·0–82·7)	73·0 (64·0–82·0)	73·5 (63·8–85·0)	<0·001
FPG (mmol/L)	5·1 (4·7–5·6)	5·0 (4·7–5·5)	5·5 (5·0–6·9)	<0·001
ALT (U/L)	21·0 (15·0–33·0)	21·0 (15·0–32·4)	25·0 (16·0–43·0)	<0·001
AST (U/L)	23·7 (19·0–31·0)	23·0 (19·0–30·1)	25·0 (19·1–34·0)	<0·001
HDL-C (mmol/L)	1·1 (0·9–1·3)	1·1 (0·9–1·3)	1·0 (0·8–1·2)	<0·001
LDL-C (mmol/L)	2·5 (2·0–3·0)	2·5 (2·0–2·9)	2·6 (2·1–3·2)	<0·001
TC (mmol/L)	4·1 (3·6–4·7)	4·1 (3·5–4·7)	4·3 (3·7–5·0)	<0·001
TG (mmol/L)	1·3 (0·9–1·8)	1·2 (0·9–1·8)	1·6 (1·1–2·3)	<0·001
HB (g/L)	140·0 (120·0–153·0)	140·0 (120·0–153·0)	138·0 (119·0–153·0)	0·099

Data are presented as n (%) for categorical variables and median (IQR) for continuous variables. p values were obtained using Pearson's χ^2^ test for categorical variables, with Fisher's exact test used when expected cell counts were small, and the Wilcoxon rank-sum test (Mann–Whitney U test) for continuous variables. Categorical variables are shown as counts with percentages in parentheses. MM, metabolic multimorbidity; IDU, injection drug use; MSM, men who have sex with men; ART, antiretroviral therapy; NNRTI, non-nucleoside reverse transcriptase inhibitor; PI/r, ritonavir-boosted protease inhibitor; INSTI, integrase strand transfer inhibitor; HBV, hepatitis B virus; HCV, hepatitis C virus; CD4, cluster of differentiation 4; HIV RNA, human immunodeficiency virus ribonucleic acid; ALT, alanine aminotransferase; AST, aspartate aminotransferase; FPG, fasting plasma glucose; HDL-C, high-density lipoprotein cholesterol; LDL-C, low-density lipoprotein cholesterol; TC, total cholesterol; TG, triglycerides; HB, haemoglobin; WBC, white blood cell.

Initial ART regimens differed by baseline MM status (*p* < 0·001). INSTI-based regimens were more frequently used among participants with MM (27·5% vs 13·3%), whereas NNRTI-based regimens were less common (58·6% vs 77·9%). In laboratory profiles, participants with baseline MM had higher FPG, ALT, and AST than those without MM (all *p* < 0·001).

### Incidence and prevalence trends of metabolic conditions from 2010 to 2024

**Fig. 1:**
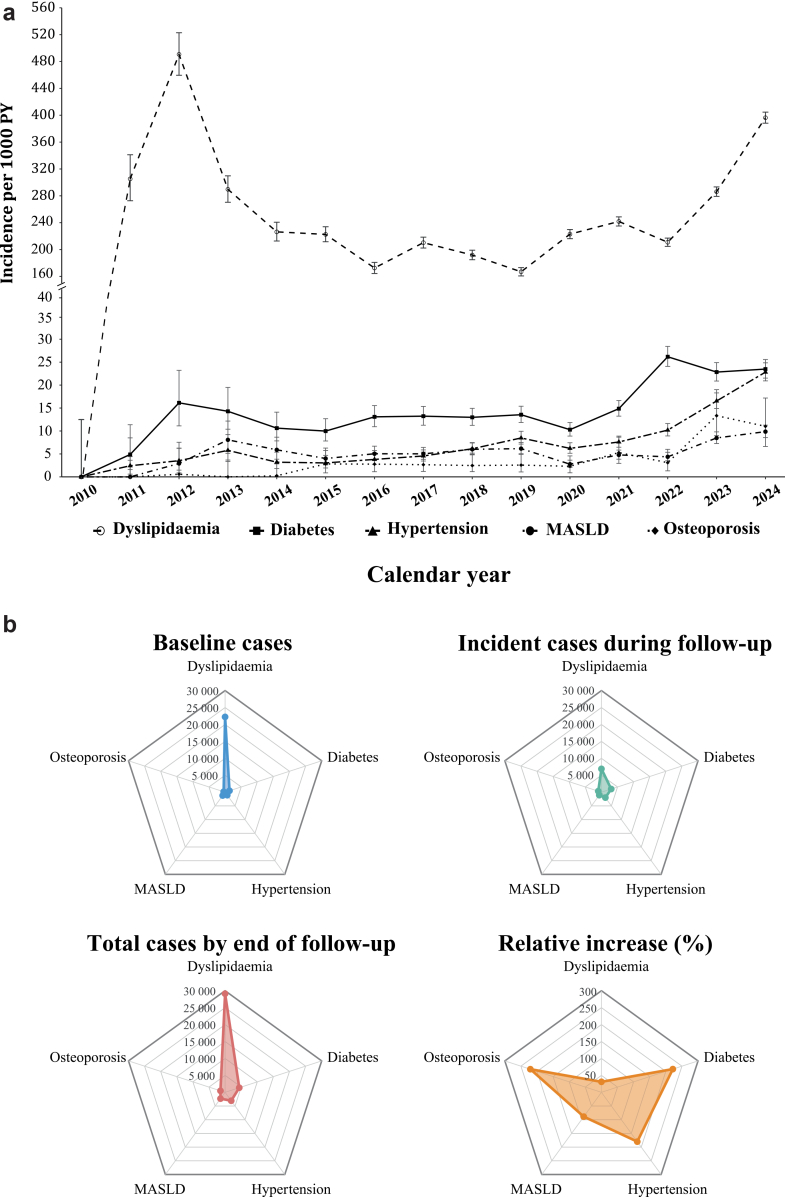
**Temporal trends in five metabolic conditions.** (a) Annual incidence rates (per 1000 PY) by calendar year, with error bars indicating 95% CI. The y-axis is truncated to improve visualisation. (b) Radar plots show baseline cases, incident cases during follow-up, and total cases by end of follow-up, and relative increase for each metabolic condition. PWH: people with HIV; MASLD: metabolic dysfunction–associated steatotic liver disease; PY: person-years; CI: confidence interval.

Annual incidence rates of all five metabolic conditions increased between 2010 and 2024, with the most pronounced rises occurring in the later years of follow-up (Fig. 1A; Supplementary Table S2). Dyslipidaemia was the most common incident metabolic condition. Its incidence was 305·3 per 1000 person-years (PY) in 2011, reached an early peak of 490·7 per 1000 PY in 2012, and then remained relatively stable at around 200 per 1000 PY for nearly a decade. From 2022 onwards, the incidence increased rapidly again, reaching 396·3 per 1000 PY in 2024.

Diabetes, hypertension, and MASLD displayed similar temporal patterns. Each showed a modest early peak in 2012–2013, followed by a relatively stable phase and then a marked increase after 2020. The incidence of diabetes increased from 10·3 per 1000 PY in 2020 to 26·2 per 1000 PY in 2022, representing an approximately 2·5-fold increase. Between 2020 and 2024, the incidence of hypertension rose from 6·2 to 22·8 per 1000 PY and MASLD from 2·8 to 9·9 per 1000 PY, with both approaching a four-fold increase relative to 2020. Osteoporosis did not show an early peak. Instead, its incidence increased steadily throughout the study period and rose more than three-fold during 2022–2023, peaking at 13·3 per 1000 PY in 2023.

Prevalence patterns broadly mirrored the incidence trends (Fig. 1B; Supplementary Table S3). Dyslipidaemia remained the most prevalent metabolic condition at baseline, during follow-up, and at the end of observation, although it exhibited the smallest relative increase (31·0%). In contrast, hypertension, diabetes, and osteoporosis showed much larger proportional increases, each exceeding 180%. Hypertension and diabetes had both appreciable baseline prevalence and substantial accumulation over time. Osteoporosis, although uncommon at baseline, increased rapidly during follow-up, and the number of incident cases eventually approached that of MASLD, whose overall prevalence increased by nearly 90%. Age-stratified analyses showed that participants older than 45 years had a substantially higher baseline prevalence of MM than those aged 45 years or younger (17·6% [1578/8946] vs 6·9% [1565/22,684]), whereas MM accumulated more rapidly during follow-up among those aged 45 years or younger (Supplementary Figures S1 and S2).

### Accumulation and patterns of MM

**Fig. 2:**
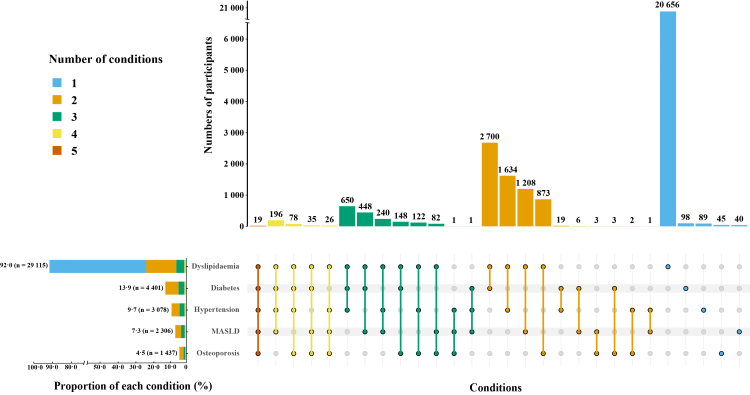
**Distribution and clustering of five metabolic conditions.** Vertical bars show the absolute number of participants with each unique combination of conditions; the y-axis is truncated to improve visualisation. Bar colours indicate the total number of conditions in that combination (1–5). Dots indicate which conditions are included in each combination, and connecting lines link conditions that co-occur. Horizontal stacked bars show, for each condition, its proportion in the total study population, together with the distribution by number of coexisting conditions; corresponding sample sizes are shown in the labels. MASLD: metabolic dysfunction–associated steatotic liver disease.

Over follow-up, the progressive accumulation of metabolic conditions resulted in diverse coexistence patterns, including both single conditions and multimorbid clusters (Fig. 2). Dyslipidaemia was the condition most likely to occur alone, accounting for 20,656 cases and 71·0% of all PWH who had dyslipidaemia. In contrast, diabetes, hypertension, MASLD, and osteoporosis rarely occurred alone, with more than 97% of cases of each coexisting with at least one additional metabolic condition.

A total of 8495 MM cases were identified, most of which involved dual–condition combinations. MM was largely driven by combinations of dyslipidaemia, diabetes, and hypertension. The most frequent dual combination was dyslipidaemia with diabetes (2700 cases; 31·8%), followed by dyslipidaemia with hypertension (1634; 19·2%). Among triple-condition clusters, the coexistence of dyslipidaemia, diabetes, and hypertension was most common (650; 7·7%). Additionally, 196 participants (2·3%) developed a four-condition combination involving dyslipidaemia, diabetes, hypertension, and MASLD, and 19 (0·2%) presented with all five metabolic conditions.

PAM clustering at 1, 3, and 5 years after ART initiation identified five recurrent metabolic profiles that were consistent with these coexistence patterns. A dyslipidaemia-dominant cluster consistently accounted for the largest subgroup, while diabetes–dyslipidaemia and dyslipidaemia–hypertension remained prominent across timepoints. Dyslipidaemia–MASLD also emerged as a recurrent cluster (Supplementary Figure S3).

### Associations of metabolic comorbidity burden and combinations with mortality

**Fig. 3:**
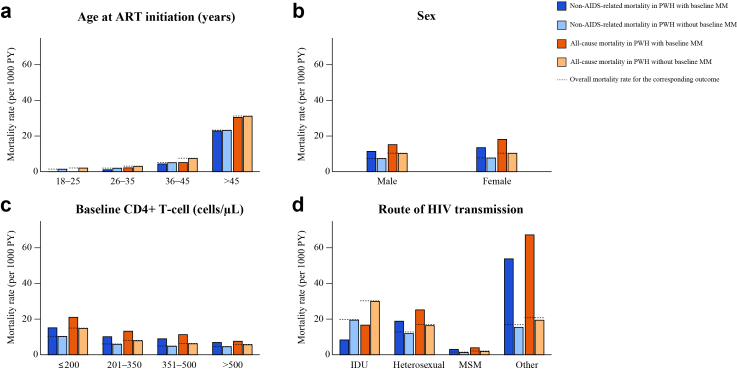
**All-cause and non–AIDS-related mortality rates by baseline MM and participant characteristics.** (a) Age at ART initiation (b) Sex (c) Baseline CD4+ T-cell count (d) Route of HIV transmission. Bars show mortality rates (per 1000 PY) for all-cause and non–AIDS-related mortality among participants with baseline MM versus those without baseline MM. Dashed horizontal lines indicate the overall mortality rate for the corresponding outcome within each subgroup, irrespective of baseline MM status. PWH, people with HIV; MM: metabolic multimorbidity; PY: person-years; ART: antiretroviral therapy; IDU, injection drug use; MSM, men who have sex with men.

Baseline MM was associated with higher all-cause and non–AIDS-related mortality across multiple subgroups (Fig. 3; Supplementary Tables S4 and S5). The distribution of AIDS-related and non–AIDS-related deaths, together with the numbers of specific causes of death, is shown in Supplementary Table S6. Compared with participants without baseline MM, those with baseline MM had approximately 1·5-fold higher mortality among both men and women. Similar patterns were observed among participants with baseline CD4+ T-cell counts of ≤500 cells/μL, among whom MM was associated with roughly 1·4–1·9-fold higher mortality compared with those without MM, whereas the differences were less pronounced in those with baseline CD4+ T-cell counts >500 cells/μL. Elevated mortality associated with baseline MM was observed both among participants infected through heterosexual contact and among MSM, but not among those infected through IDU. Absolute mortality risks increased with age for both all-cause and non–AIDS-related deaths, with the highest rates observed among individuals aged >45 years regardless of baseline MM status. For metabolic-related mortality, however, participants older than 45 years with baseline MM had higher mortality rates than those without baseline MM (Supplementary Figure S4).

**Fig. 4:**
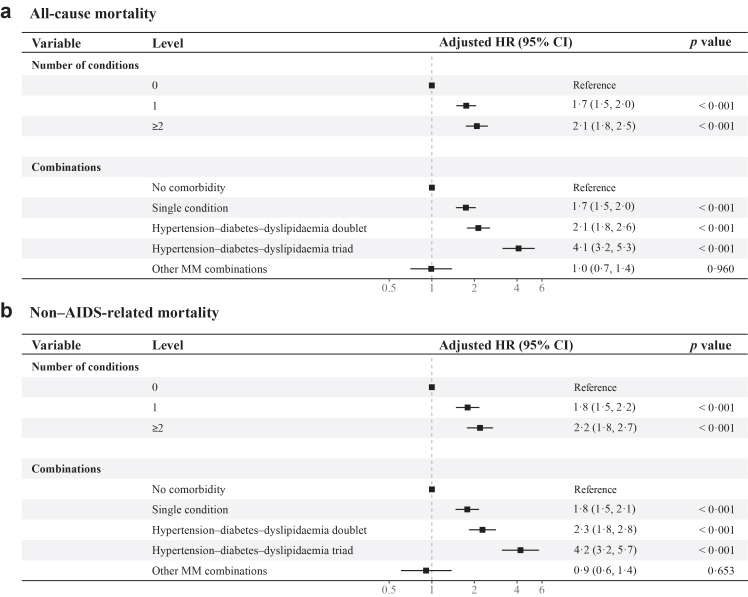
**Adjusted HRs of all-cause mortality and non–AIDS-related mortality by number of conditions and MM combinations.** (a) All-cause mortality (b) Non–AIDS-related mortality. “Hypertension–diabetes–dyslipidaemia doublet” refers to any two-condition combination among hypertension, diabetes, and dyslipidaemia. “Hypertension–diabetes–dyslipidaemia triad” refers to the coexistence of hypertension, diabetes, and dyslipidaemia. Adjusted HRs with 95% CIs were estimated using Cox regression models with time-dependent MM. One model used metabolic comorbidity counts (0, 1, and ≥2 conditions), and the other used MM combinations (no comorbidity, single condition, hypertension–diabetes–dyslipidaemia doublet, hypertension–diabetes–dyslipidaemia triad, and other MM combinations) as the primary exposure, with the no comorbidity group as the reference. Both models were adjusted for age at ART initiation, sex, marital status, route of HIV transmission, baseline CD4+ T-cell count, baseline HIV RNA viral load, initial ART regimen, HBV/HCV co-infection status, and routine laboratory indicators (e.g., white blood cell count, platelet count, creatinine, fasting plasma glucose, alanine aminotransferase, aspartate aminotransferase, high-density lipoprotein cholesterol, low-density lipoprotein cholesterol, total cholesterol, and triglycerides). HR, hazard ratio; MM, metabolic multimorbidity; PWH, people with HIV; ART, antiretroviral therapy; HBV, hepatitis B virus; HCV, hepatitis C virus; CI: confidence interval.

When the number of metabolic conditions was modelled as a time-varying exposure, a clear dose–response relationship between metabolic comorbidity burden and mortality was observed (Fig. 4, Supplementary Table S7). All-cause mortality rates were 7·2 (95% CI 6·2–8·3), 10·3 (95% CI 9·7–10·8), and 15·9 (95% CI 14·5–17·4) per 1000 PY among participants with 0, 1 and ≥ 2 conditions, respectively, corresponding to an approximately 1·5-fold increase with each additional metabolic condition. Compared with participants without metabolic comorbidity, those with one condition had nearly a two-fold higher risk of all-cause mortality (aHR 1·7; 95% CI 1·5–2·0), whereas those with ≥2 conditions had a more than two-fold higher risk (aHR 2·1; 95% CI 1·8–2·5). Associations were similar for non–AIDS-related mortality, with corresponding aHRs of 1·8 (95% CI 1·5–2·2) and 2·2 (95% CI 1·8–2·7) for one and ≥2 conditions, respectively.

Mortality risks also varied substantially according to specific MM combinations (Fig. 4, Supplementary Table S7). Participants with a hypertension–diabetes–dyslipidaemia doublet had an all-cause mortality rate of 18·1 (95% CI 16·2–20·1) per 1000 PY, whereas those with the corresponding triad had a rate of 43·0 (95% CI 34·9–52·4) per 1000 PY, representing approximately 2·5-fold and six-fold higher rates, respectively, than those without metabolic comorbidity. In adjusted models, the hypertension–diabetes–dyslipidaemia doublet and triad were associated with aHRs of 2·1 (95% CI 1·8–2·6) and 4·1 (95% CI 3·2–5·3) for all-cause mortality, respectively. The corresponding aHRs for non–AIDS-related mortality were 2·3 (95% CI 1·8–2·8) and 4·2 (95% CI 3·2–5·7). Similar patterns were observed for metabolic-related mortality. Compared with no metabolic comorbidity, ≥2 metabolic conditions were associated with an aHR of 2·5 (95% CI 1·7–3·8), while the hypertension–diabetes–dyslipidaemia doublet and triad were associated with aHRs of 2·6 (95% CI 1·7–4·0) and 5·1 (95% CI 3·0–8·8) for metabolic-related mortality, respectively (Supplementary Tables S8 and S9). In age-stratified analyses of all-cause mortality, the associations between MM and mortality were more pronounced among participants who initiated ART at age older than 45 years, whereas among those aged 45 years or younger, elevated risk was largely confined to the hypertension–diabetes–dyslipidaemia triad (Supplementary Table S10).

In additional analyses of non-fatal outcomes, MM was also associated with incident CKD and CVD (Supplementary Table S11). The associations were stronger for CKD, particularly among participants with ≥2 metabolic conditions (adjusted sHR 6·2, 95% CI 3·7–10·4) and those with the hypertension–diabetes–dyslipidaemia triad (adjusted sHR 15·5, 95% CI 8·4–28·6). For CVD, the highest excess risk was likewise observed for the triad (adjusted sHR 2·1, 95% CI 1·6–2·8).

Although proportional hazards diagnostics indicated some departures from the proportional hazards assumption for MM exposures, piecewise Cox analyses were broadly consistent with the primary results. These analyses showed time-varying but directionally stable associations across follow-up intervals for ≥2 metabolic conditions and for the hypertension–diabetes–dyslipidaemia doublet and triad (Supplementary Tables S12–S14; Supplementary Figure S5). Accordingly, the overall aHRs reported above should be interpreted as average associations over follow-up rather than as constant effects over time. Full-model estimates for all covariates are provided in Supplementary Tables S15–S18. The main associations were materially unchanged in complete-case analyses and in sensitivity analyses using a 180-day rather than 90-day lag period (Supplementary Tables S19 and S20).

## Discussion

In this large two-centre cohort of PWH initiating ART in southern China, MM became increasingly common over time. Incidence rates of all major metabolic conditions increased during follow-up, with particularly marked rises after 2020. Most multimorbidity patterns were centred on dyslipidaemia, diabetes and hypertension, and the hypertension–diabetes–dyslipidaemia doublet and triad were associated with substantially higher risks of mortality. MM burden also showed a clear dose–response relationship with mortality when modelled as a time-varying exposure. Together, these findings highlight the growing importance of metabolic health in contemporary HIV care and support the need for integrated long-term management strategies in the ART era.

MM was already apparent at ART initiation, suggesting that metabolic burden was not merely a late consequence of long-term therapy but also an early feature of contemporary HIV care among PWH. Older age remained an important determinant of multimorbidity, whereas HIV-specific characteristics differed only modestly at baseline. Baseline ART regimens also varied by MM status, with higher use of INSTI-based therapy and lower use of NNRTI-based regimens, which may partly reflect regimen selection at ART initiation. In an observational setting, this difference may reflect both confounding by indication, whereby treatment was selected according to underlying metabolic risk, and the possibility of treatment-related metabolic effects associated with specific ART classes. Against this baseline context, the progressive rise in metabolic conditions in our cohort is broadly consistent with reports from high-income settings and emerging cohorts in sub-Saharan Africa, where dyslipidaemia, hypertension, and diabetes have become increasingly common among PWH receiving long-term ART.^23^, ^24^, ^25^ Our study extends existing evidence by providing a 15-year longitudinal perspective and showing a pronounced acceleration in the incidence of multiple metabolic conditions after 2020. This pattern may reflect the combined influence of population ageing, persistent immune activation despite viral suppression, and increasing exposure to contemporary ART regimens.^14^^,^^26^^,^^27^ The sharp increases in MASLD and osteoporosis highlight the expanding spectrum of metabolic dysfunction in PWH and warrant closer attention in those receiving long-term ART.

The increase in metabolic conditions after 2020 may also reflect the broader repercussions of the COVID-19 pandemic. This pattern may be related not only to lifestyle disruptions, including reduced physical activity, dietary shifts, and increased sedentary behaviour, but also to delays in diagnosis and shifts in case ascertainment during the pandemic.^28^, ^29^, ^30^ Evidence from China further suggests that HIV testing, treatment initiation, and continuity of care were affected during this period.^31^^,^^32^ Among PWH, these overlapping disruptions may have contributed to the greater metabolic burden observed in the later years of follow-up.

The strong associations between metabolic comorbidities and mortality observed in this cohort are consistent with previous studies showing that these conditions contribute substantially to the rising burden of non–AIDS-related deaths among PWH in the ART era. The limited separation in overall non–AIDS-related mortality between baseline MM groups among participants older than 45 years may reflect the broad and heterogeneous nature of this outcome, which includes causes not necessarily directly related to the metabolic conditions under study and may therefore attenuate MM-related differences in older adults who already have a high background mortality risk. Earlier cohorts have documented elevated cardiometabolic risk among individuals with hypertension, diabetes or dyslipidaemia; however, reliance on baseline comorbidity status limited their ability to capture time-dependent metabolic changes.^33^ By modelling metabolic comorbidities as a time-varying exposure, our study provides more robust evidence of a dose–response relationship between cumulative metabolic comorbidities burden and mortality. In addition, the identification of particularly high–risk combinations, especially the hypertension–diabetes–dyslipidaemia doublet and triad, provides further insight into the combinations of metabolic conditions associated with the greatest vulnerability to non–AIDS-related death. These associations are biologically plausible and consistent with shared pathways involving chronic inflammation, endothelial dysfunction, accelerated atherosclerosis, and ART-related metabolic effects, which may jointly amplify cardiovascular and hepatic risk.^34^, ^35^, ^36^

Metabolic comorbidities become more common with advancing age, whereas HIV may be acquired at different stages of the life course. In this context, the metabolic burden in PWH might develop through two broad ageing-related trajectories.^37^ One involves acquiring HIV at an older age, as reflected by the higher baseline burden of MM among participants older than 45 years, in whom age-related metabolic conditions might already be established at treatment initiation, continue to accumulate during follow-up, and be more strongly associated with all-cause mortality. In these individuals, acquisition of HIV infection and subsequent initiation of ART might act as additional stressors superimposed on pre-existing metabolic vulnerability.^14^^,^^26^^,^^27^ The other trajectory involves acquiring HIV earlier in adulthood and ageing with HIV over time. Although individuals younger than 45 in our study had a lower baseline burden of MM, metabolic conditions accumulated rapidly during follow-up; however, their associations with mortality were less marked. Together, these findings suggest that the clinical implications of MM may depend not only on the extent of metabolic burden itself, but also on the stage of life at which individuals acquire HIV and enter care. This distinction highlights the importance of a life-course perspective in metabolic risk assessment and supports more age-contextualised monitoring and prevention strategies in PWH.

Our findings have important implications for clinical practice and public health. First, as MM has become an important determinant of mortality among PWH, HIV care models that have historically focused mainly on virological suppression need to be broadened to systematically incorporate the prevention, early detection, and long-term management of metabolic comorbidities. Dyslipidaemia, hypertension, diabetes, MASLD, and osteoporosis should be routinely assessed from the early stages of ART and monitored longitudinally to identify individuals at risk of accumulating MM, with particular attention to osteoporosis onset and progression in older adults and long-term ART users. Second, our results also support the incorporation of MM-based risk stratification into HIV care. Considering both the number of metabolic conditions and the presence of high-risk MM combinations (e.g., hypertension–diabetes–dyslipidaemia doublet or triad) may help identify individuals who require more intensive monitoring, closer follow-up, and prioritised access to cardiometabolic risk reduction interventions. The clustering of cardiovascular, hepatic, and bone-related conditions underscores the need for closer collaboration between HIV services and relevant specialities such as cardiology, endocrinology, hepatology, and orthopaedics. In addition, prioritising long-term metabolic safety when initiating or modifying ART, alongside structured lifestyle interventions, may help to attenuate the accumulation of metabolic comorbidities and reduce its downstream impact on morbidity and mortality.

Several limitations should be considered when interpreting our findings. First, this study was conducted at two HIV care centres in southern China, which may limit the representativeness of the cohort and the applicability of the findings to settings with different population characteristics, care patterns, and metabolic risk profiles. Such differences across settings may also affect the observed associations between MM and mortality. Second, metabolic comorbidities were ascertained from diagnoses or ICD-10 codes, supported by available laboratory measurements. Non-uniform screening across centres and over time may have led to under-ascertainment and misclassification. Our predefined categorisation of metabolic combinations might also not have fully captured the heterogeneity of multimorbidity, particularly without data on disease severity, duration, and treatment control. Third, cause-of-death information for out-of-hospital deaths was sometimes less granular, limiting more detailed subclassification of non–AIDS-related causes. Fourth, differences in ART class use at baseline and the increase in metabolic comorbidities during follow-up may partly reflect underlying clinical decision-making, ageing, calendar effects, and changes in clinical practice, and should not be attributed solely to ART initiation. Finally, key lifestyle and socioeconomic factors, such as smoking, alcohol use, diet, physical activity, and income, were not systematically captured in clinical records, and detailed information on baseline non-metabolic comorbidities was limited. Therefore, residual unmeasured confounding remains possible, and although the time-varying modelling approach reduced simultaneity bias, reverse causality cannot be fully excluded.

### Conclusion

In this large longitudinal cohort study of PWH in southern China, metabolic comorbidities became increasingly common over follow-up, contributing to a growing burden of MM. Cumulative MM burden and specific high–risk combinations, particularly combinations of dyslipidaemia, diabetes, and hypertension, were strongly associated with high risks of all-cause and non–AIDS-related mortality. These findings highlight metabolic health as an important determinant of long-term outcomes in the contemporary ART era and underscore the need for HIV care models that integrate systematic assessment and longitudinal management of MM.

## Contributors

CL, LS, and JL contributed to the conception of the study. LS, YH, FZ, JH, JW, and YLi directly accessed and verified the underlying data. XL, YLuo, DZ, and XX performed the preliminary data analysis and data curation. CL, XX, and JL developed the methodology. CL drafted the initial manuscript. JL, HW, HL, and PC reviewed and revised the manuscript. CL and JL made the final decision to submit the manuscript for publication. All authors had full access to the data in the study and approved the final version of the manuscript.

## Data sharing statement

The data used in this study involve PWH and are therefore confidential. Individual-level data cannot be shared publicly due to privacy protection and ethical restrictions. Reasonable requests for access to deidentified data may be directed to the corresponding author, subject to approval by the institutional ethics committee.

## Declaration of interests

The authors declare that they have no competing interests.
