# Supplementary material for: Accumulation of metabolic multimorbidity and its association with mortality: results from a prospective cohort study among people with HIV in China, 2010–2024
Source: Lancet Reg Health West Pac. 2026 May 22;70:101883. doi: 10.1016/j.lanwpc.2026.101883 (PMC13218219; doi:10.1016/j.lanwpc.2026.101883)
Supplement: Supplementary Materials [file mmc1.docx]

**Supplementary Materials**

**Accumulation of metabolic multimorbidity and its association with mortality: results from a prospective cohort study among people with HIV in China, 2010–2024**

1. ***Brief Introduction of the Cohort*** 1
2. ***Supplementary Table 1.*** *ICD-10 codes and diagnostic criteria for five metabolic comorbidities* 2
3. ***Supplementary Table 2.*** *Annual incidence rates of five metabolic conditions, 2010–2024* 3
4. ***Supplementary Table 3.*** *Baseline prevalence and increase over time for the five individual metabolic conditions of interest* 4
5. ***Supplementary Table 4.*** *Mortality rates by baseline MM status and subgroup* 5
6. ***Supplementary Table 5.*** *Mortality rate ratios comparing baseline MM vs no MM by subgroup* 7
7. ***Supplementary Table 6.*** *Distribution of specific causes of death (non–AIDS-related, AIDS-related, and undetermined), and classification of metabolic-related causes among non–AIDS-related deaths* 9
8. ***Supplementary Table 7.*** *Relationship between numbers and combinations of metabolic conditions, modelled as time-varying exposure, and all-cause (top part) and non-AIDS related (bottom part) mortality* 11
9. ***Supplementary Table 8.*** *Relationship between numbers of metabolic conditions, modelled as time-varying exposure, and metabolic-related mortality* 13
10. ***Supplementary Table 9.*** *Relationship between combinations of metabolic conditions, modelled as time-varying exposure, and metabolic-related mortality* 15
11. ***Supplementary Table 10****. Relationship between numbers of metabolic conditions, modelled as time-varying exposure, and all-cause mortality, by age at time of ART initiation* 17
12. ***Supplementary Table 11.*** *Adjusted subdistribution hazard ratios of incident CKD and CVD in relation to the numbers and combinations of metabolic comorbidities* 19
13. ***Supplementary Table 12****. Tests of the proportional hazards assumption for covariates in the Cox regression model with MM parameterised by number of conditions* 21
14. ***Supplementary Table 13.*** *Tests of the proportional hazards assumption for covariates in the Cox regression model with MM parameterised by combinations* 22
15. ***Supplementary Table 14.*** *Time-stratified adjusted hazard ratios for all-cause mortality and non–AIDS-related mortality according to number of conditions and MM combinations* 23
16. ***Supplementary Table 15.*** *Relationship between numbers of metabolic conditions, modelled as time-varying exposure, and other covariates with all-cause mortality* 27
17. ***Supplementary Table 16.*** *Relationship between combinations of metabolic conditions, modelled as time-varying exposure, and other covariates with all-cause mortality* 29
18. ***Supplementary Table 17.*** *Relationship between numbers of metabolic conditions, modelled as time-varying exposure, and other covariates with non–AIDS-related mortality* 31
19. ***Supplementary Table 18.*** *Relationship between combinations of metabolic conditions, modelled as time-varying exposure, and other covariates with non-AIDS-related mortality* 33
20. ***Supplementary Table 19.*** *Complete-case sensitivity analysis of the associations of the number of metabolic conditions and combinations with all-cause and non–AIDS-related mortality* 35
21. ***Supplementary Table 20.*** *Relationship between numbers and combinations of metabolic conditions, modelled as time-varying exposure with a 180-day lag time after MM onset, and all-cause mortality* 37
22. ***Supplementary Figure 1.*** *Radar plots of metabolic condition burden among participants aged ≤45 years* 38
23. ***Supplementary Figure 2.*** *Radar plots of metabolic condition burden among participants aged >45 years* 39
24. ***Supplementary Figure 3.*** *Exploratory clustering of metabolic multimorbidity patterns at 1, 3, and 5 years after ART initiation* 40
25. ***Supplementary Figure 4.*** *Metabolic-related mortality rates by baseline MM and participant characteristics* 41
26. ***Supplementary Figure 5.*** *Scaled Schoenfeld residuals for assessing proportional hazards assumption in Cox models* 42

***Brief Introduction of the Cohort***

This study was conducted in two major HIV treatment centers in southern China: the Third People’s Hospital of Shenzhen, the designated hospital for HIV care in Shenzhen, and the Fourth People’s Hospital of Nanning, the largest specialized center for HIV management in Guangxi Province. Together, these hospitals managed more than 36 000 people with HIV (PWH) between 2010 and 2024. Antiretroviral therapy (ART) has been provided free of charge in both sites in accordance with national HIV treatment guidelines. Before ART initiation, all patients undergo comprehensive baseline assessments, including HIV viral load, CD4 and CD8 T-cell counts, screening for common opportunistic infections, routine blood and biochemical tests (liver, renal, and thyroid function), electrocardiography, bone density testing, and screening for co-infections such as cytomegalovirus, hepatitis B virus, hepatitis C virus, syphilis, and human papillomavirus. Detailed medical history and lifestyle information are also recorded. Following ART initiation, patients are scheduled for follow-up visits every three months, which include routine viral load and T-cell subset monitoring, supplemented with additional examinations as clinically indicated. The high reimbursement coverage of local health insurance systems in both Shenzhen and Nanning has contributed to low rates of loss to follow-up, ensuring a large and stable longitudinal cohort with high-quality clinical data for epidemiological research.

***Supplementary Table 1. ICD-10 codes and diagnostic criteria for five metabolic comorbidities***

| **Comorbidity** | **ICD-10** | **Clinical and laboratory diagnostic criteria** |
| --- | --- | --- |
| Dyslipidaemia | E78 | Any one of the following: TC ≥5·2 mmol/L, HDL-C <1·0 mmol/L, LDL-C ≥3·4 mmol/L, or TG ≥1·7 mmol/L |
| Hypertension | I10–I15 | Blood pressure ≥140/90 mmHg |
| Diabetes mellitus | E10–E14 | HbA1c ≥6·5%, fasting plasma glucose ≥7·0 mmol/L, or use of antidiabetic medications, or a self-reported history of diabetes |
| Metabolic dysfunction-associated steatotic liver disease (MASLD) | K76·0 | Imaging evidence of hepatic steatosis plus at least one metabolic risk factor (overweight/obesity, T2DM, dyslipidaemia, hypertension, etc.), and exclusion of significant alcohol use or viral hepatitis |
| Osteoporosis | M80–M82 | Bone mineral density T-score ≤ -2·5 |

ICD-10, International Classification of Diseases, 10th Revision; TC, total cholesterol; HDL-C, high-density lipoprotein cholesterol; LDL-C, low-density lipoprotein cholesterol; TG, triglycerides; HbA1c, glycated hemoglobin; T2DM, type 2 diabetes mellitus.

***Supplementary Table 2. Annual incidence rates of five metabolic conditions, 2010–2024***

| **Year** | **Condition (per 1000 PY)** | | | | | | | | | **Participants under follow-up, N** |
| --- | --- | --- | --- | --- | --- | --- | --- | --- | --- | --- |
|  | **Dyslipidaemia** |  | **Diabetes** |  | **Hypertension** |  | **MASLD** |  | **Osteoporosis** |  |
| 2010 | 0·0 |  | 0·0 |  | 0·0 |  | 0·0 |  | 0·0 | 620 |
| 2011 | 305·3 |  | 4·9 |  | 2·4 |  | 0·0 |  | 0·0 | 1539 |
| 2012 | 490·7 |  | 16·1 |  | 3·5 |  | 2·9 |  | 0·6 | 2324 |
| 2013 | 289·7 |  | 14·3 |  | 5·8 |  | 8·1 |  | 0·0 | 3738 |
| 2014 | 226·3 |  | 10·6 |  | 3·2 |  | 5·9 |  | 0·2 | 5769 |
| 2015 | 222·6 |  | 10·0 |  | 3·0 |  | 4·0 |  | 2·8 | 8909 |
| 2016 | 172·5 |  | 13·1 |  | 3·8 |  | 5·0 |  | 2·8 | 11933 |
| 2017 | 210·3 |  | 13·2 |  | 4·5 |  | 5·0 |  | 2·7 | 14718 |
| 2018 | 191·9 |  | 13·0 |  | 6·1 |  | 6·0 |  | 2·5 | 17262 |
| 2019 | 166·9 |  | 13·6 |  | 8·5 |  | 6·2 |  | 2·6 | 19474 |
| 2020 | 223·0 |  | 10·3 |  | 6·2 |  | 2·8 |  | 2·3 | 21263 |
| 2021 | 241·8 |  | 14·9 |  | 7·6 |  | 4·8 |  | 5·3 | 23134 |
| 2022 | 210·8 |  | 26·2 |  | 10·2 |  | 4·4 |  | 3·1 | 24328 |
| 2023 | 286·2 |  | 22·8 |  | 16·5 |  | 8·5 |  | 13·3 | 25437 |
| 2024 | 396·3 |  | 23·5 |  | 22·8 |  | 9·9 |  | 11·0 | 25625 |

PWH, people with HIV; PY, person-years; MASLD, metabolic dysfunction–associated steatotic liver disease. Annual incidence rates (per 1000 PY) were calculated as the number of incident cases divided by total person-years for each calendar year. Incident cases were defined as new diagnoses occurring ≥ 90 days after ART initiation. This table provides the data used to construct Figure 1A.

***Supplementary Table 3. Baseline prevalence and increase over time for the five individual metabolic conditions of interest***

| **Condition** | **Baseline cases** | **Incident cases during follow-up** | **Total cases by end of follow-up** | **Relative increase (%)** |
| --- | --- | --- | --- | --- |
| Dyslipidaemia | 22 230 | 6 885 | 29 115 | 31·0 |
| Diabetes | 1 368 | 3 033 | 4 401 | 221·7 |
| Hypertension | 1 099 | 1 979 | 3 078 | 180·1 |
| MASLD | 1 221 | 1 088 | 2 306 | 88·9 |
| Osteoporosis | 448 | 989 | 1 437 | 220·8 |

MASLD, metabolic dysfunction–associated steatotic liver disease. Baseline cases were defined as diagnoses occurring within 90 days after ART initiation; incident cases were defined as new diagnoses occurring ≥90 days after ART initiation. Total cases by end of follow-up represent the cumulative number of affected individuals. Relative increase (%) was calculated as incident cases during follow-up divided by baseline cases × 100%. This table provides the data used to construct Figure 1B.

***Supplementary Table 4. Mortality rates by baseline MM status and subgroup***

| **Baseline status** | **Factor: Level** | **PY** | **All-cause mortality** | **All-cause mortality rate** | **Non-AIDS-related mortality** | **Non-AIDS-related mortality rate** |
| --- | --- | --- | --- | --- | --- | --- |
| **MM** | Age at ART initiation (years): 18–25 | 693·7 | 0 | 0·0 | 0 | 0·0 |
|  | Age at ART initiation (years): 26–35 | 3 726·3 | 8 | 2·2 | 4 | 1·1 |
|  | Age at ART initiation (years): 36–45 | 3 903·2 | 20 | 5·1 | 17 | 4·4 |
|  | Age at ART initiation (years): >45 | 6 827·9 | 208 | 30·5 | 156 | 22·9 |
|  | Sex: Male | 12 999·8 | 197 | 15·2 | 148 | 11·4 |
|  | Sex: Female | 2 151·4 | 39 | 18·1 | 29 | 13·5 |
|  | Baseline CD4+ T-cell (cells/µL): ≤200 | 6 283·8 | 132 | 21·0 | 95 | 15·1 |
|  | Baseline CD4+ T-cell (cells/µL): 201–350 | 4 846·4 | 64 | 13·2 | 49 | 10·1 |
|  | Baseline CD4+ T-cell (cells/µL): 351–500 | 2 568·7 | 29 | 11·3 | 23 | 9·0 |
|  | Baseline CD4+ T-cell (cells/µL): >500 | 1 452·2 | 11 | 7·6 | 10 | 6·9 |
|  | Route of HIV transmission: IDU | 120·3 | 2 | 16·6 | 1 | 8·3 |
|  | Route of HIV transmission: Heterosexual | 8 025·3 | 202 | 25·2 | 151 | 18·8 |
|  | Route of HIV transmission: MSM | 6 931·3 | 27 | 3·9 | 21 | 3·0 |
|  | Route of HIV transmission: Other | 74·4 | 5 | 67·2 | 4 | 53·8 |
| **No MM** | Age at ART initiation (years): 18–25 | 28 354·4 | 57 | 2·0 | 39 | 1·4 |
|  | Age at ART initiation (years): 26–35 | 70 027·7 | 210 | 3·0 | 134 | 1·9 |
|  | Age at ART initiation (years): 36–45 | 40 628·3 | 303 | 7·5 | 204 | 5·0 |
|  | Age at ART initiation (years): >45 | 41 059·6 | 1 277 | 31·1 | 951 | 23·2 |
|  | Sex: Male | 146 989·2 | 1 506 | 10·3 | 1 075 | 7·3 |
|  | Sex: Female | 33 080·8 | 341 | 10·3 | 253 | 7·7 |
|  | Baseline CD4+ T-cell (cells/µL): ≤200 | 74 136·2 | 1 096 | 14·8 | 758 | 10·2 |
|  | Baseline CD4+ T-cell (cells/µL): 201–350 | 62 975·1 | 494 | 7·8 | 369 | 5·9 |
|  | Baseline CD4+ T-cell (cells/µL): 351–500 | 30 262·2 | 186 | 6·2 | 144 | 4·8 |
|  | Baseline CD4+ T-cell (cells/µL): >500 | 12 696·4 | 71 | 5·6 | 57 | 4·5 |
|  | Route of HIV transmission: IDU | 4 380·2 | 131 | 29·9 | 85 | 19·4 |
|  | Route of HIV transmission: Heterosexual | 94 074·3 | 1 536 | 16·3 | 1 119 | 11·9 |
|  | Route of HIV transmission: MSM | 79 860·9 | 146 | 1·8 | 97 | 1·2 |
|  | Route of HIV transmission: Other | 1 754·5 | 34 | 19·4 | 27 | 15·4 |

MM, metabolic multimorbidity; PY, person-years; IDU, injection drug use; MSM, men who have sex with men. Mortality rates are presented as deaths per 1000 PY. This table provides the data used to construct Figure 3.

***Supplementary Table 5. Mortality rate ratios comparing baseline MM vs no MM by subgroup***

| **Category** | **Factor: Level** | **Mortality rate ratio (95% CI)** | ***p* value** |
| --- | --- | --- | --- |
| All-cause mortality | Age at ART initiation (years): 18–25 | – | – |
|  | Age at ART initiation (years): 26–35 | 0·7 (0·3–1·4) | 0·439 |
|  | Age at ART initiation (years): 36–45 | 0·7 (0·4–1·1) | 0·115 |
|  | Age at ART initiation (years): >45 | 1·0 (0·8–1·1) | 0·824 |
|  | Sex: Male | 1·5 (1·4–1·7) | < 0·001 |
|  | Sex: Female | 1·8 (1·2–2·5) | 0·002 |
|  | Baseline CD4+ T-cell (cells/µL): ≤200 | 1·4 (1·2–1·7) | < 0·001 |
|  | Baseline CD4+ T-cell (cells/µL): 201–350 | 1·7 (1·3–2·2) | < 0·001 |
|  | Baseline CD4+ T-cell (cells/µL): 351–500 | 1·8 (1·2–2·7) | 0·005 |
|  | Baseline CD4+ T-cell (cells/µL): >500 | 1·4 (0·7–2·6) | 0·360 |
|  | Route of HIV transmission: IDU | 0·6 (0·1–2·1) | 0·591 |
|  | Route of HIV transmission: Heterosexual | 1·5 (1·3–1·8) | < 0·001 |
|  | Route of HIV transmission: MSM | 2·1 (1·4–3·2) | 0·001 |
|  | Route of HIV transmission: Other | 3·5 (1·1–8·9) | 0·020 |
| Non-AIDS-related mortality | Age at ART initiation (years): 18–25 | – | – |
|  | Age at ART initiation (years): 26–35 | 0·6 (0·2–1·5) | 0·330 |
|  | Age at ART initiation (years): 36–45 | 0·9 (0·5–1·4) | 0·720 |
|  | Age at ART initiation (years): >45 | 1·0 (0·8–1·2) | 0·931 |
|  | Sex: Male | 1·6 (1·3–1·9) | < 0·001 |
|  | Sex: Female | 1·8 (1·2–2·6) | 0·006 |
|  | Baseline CD4+ T-cell (cells/µL): ≤200 | 1·5 (1·2–1·8) | < 0·001 |
|  | Baseline CD4+ T-cell (cells/µL): 201–350 | 1·7 (1·3–2·3) | < 0·001 |
|  | Baseline CD4+ T-cell (cells/µL): 351–500 | 1·9 (1·2–2·9) | 0·009 |
|  | Baseline CD4+ T-cell (cells/µL): >500 | 1·5 (0·7–3·0) | 0·223 |
|  | Route of HIV transmission: IDU | 0·4 (0·0–2·5) | 0·732 |
|  | Route of HIV transmission: Heterosexual | 1·6 (1·3–1·9) | < 0·001 |
|  | Route of HIV transmission: MSM | 2·5 (1·5–4·0) | < 0·001 |
|  | Route of HIV transmission: Other | 3·5 (0·9–10·0) | 0·036 |

RR, rate ratio; CI, confidence interval; PY, person-years; MM, metabolic multimorbidity; IDU, injection drug use; MSM, men who have sex with men. RRs compare mortality rates in individuals with baseline MM versus those without MM within each stratum. RRs, 95% CIs, and *p* values were calculated using exact Poisson methods based on person-time. A dash (–) indicates that estimates could not be computed due to zero events in the MM group. This table provides the data used to construct Figure 3

***Supplementary Table 6. Distribution of specific causes of death (non–AIDS-related, AIDS-related, and undetermined), and classification of metabolic-related causes among non–AIDS-related deaths***

| **Cause category** | **N** | **Proportion, %** | **Metabolic-related** |
| --- | --- | --- | --- |
| **Non–AIDS-related mortality** | 1505 | 100·0 | / |
| Respiratory diseases | 313 | 20·8 | No |
| Cardio- and cerebrovascular diseases | 297 | 19·7 | Yes |
| Cancers | 230 | 15·3 | No |
| Other non–AIDS-related disease deaths | 124 | 8·2 | No |
| Other digestive system diseases | 53 | 3·5 | No |
| Other non-disease external causes of death (injury, etc.) | 48 | 3·2 | No |
| Endocrine, nutritional and metabolic diseases | 43 | 2·9 | Yes |
| Suicide | 19 | 1·3 | No |
| Drug overdose | 15 | 1·0 | No |
| Hepatitis C or hepatitis B infection | 10 | 0·7 | No |
| Adverse drug reactions | 2 | 0·1 | No |
| Unclassified non–AIDS-related cause | 351 | 23·3 | No |
| **AIDS-related mortality** | 304 | 100·0 | / |
| Other specified AIDS-related diseases and syndromes | 68 | 22·4 | NA |
| Pulmonary tuberculosis (Mycobacterium tuberculosis infection) | 36 | 11·8 | NA |
| Recurrent bacterial pneumonia | 27 | 8·9 | NA |
| Acute HIV infection syndrome | 27 | 8·9 | NA |
| Pneumocystis jirovecii pneumonia (PJP), also known as Pneumocystis pneumonia (PCP) | 26 | 8·6 | NA |
| Multiple malignant neoplasms (metastatic cancer; lymphoma not otherwise specified) | 13 | 4·3 | NA |
| Wasting syndrome | 11 | 3·6 | NA |
| Other AIDS-related malignancies | 9 | 3·0 | NA |
| Extrapulmonary tuberculosis | 8 | 2·6 | NA |
| Candidiasis (oesophageal or pulmonary/tracheobronchial) | 5 | 1·6 | NA |
| HIV encephalopathy (AIDS dementia complex, ADC) | 4 | 1·3 | NA |
| Recurrent non-typhoidal Salmonella septicaemia | 4 | 1·3 | NA |
| Disseminated or extrapulmonary coccidioidomycosis | 3 | 1·0 | NA |
| Herpes simplex virus infection (orolabial, genital, or anorectal) | 2 | 0·7 | NA |
| Chronic intestinal cryptosporidiosis (diarrhoea >1 month) | 2 | 0·7 | NA |
| Toxoplasmosis of the brain (cerebral toxoplasmosis) | 1 | 0·3 | NA |
| Cytomegalovirus disease (other than liver, spleen, or lymph nodes) | 1 | 0·3 | NA |
| Kaposi’s sarcoma | 1 | 0·3 | NA |
| Other non-Hodgkin lymphoma (primary CNS or B-cell) | 1 | 0·3 | NA |
| Cryptococcosis (cryptococcal meningitis or extrapulmonary cryptococcosis) | 1 | 0·3 | NA |
| Unclassified AIDS-related cause | 54 | 17·8 | NA |
| **Undetermined mortality cause** | 274 | 100·0 | / |

NA, not applicable; PJP, Pneumocystis jirovecii pneumonia; PCP, Pneumocystis pneumonia; ADC, AIDS dementia complex; CNS, central nervous system. Data are presented as number (N) and proportion (%), with proportions calculated within each major category (non–AIDS-related mortality, AIDS-related mortality, and undetermined mortality cause), respectively. “Metabolic-related” indicates causes of death considered potentially attributable to one or more of the five metabolic comorbidities assessed in this study; cardio- and cerebrovascular diseases and endocrine, nutritional and metabolic diseases were classified as metabolic-related, and all other cause categories were classified as non–metabolic-related. “Unclassified non–AIDS-related cause” and “Unclassified AIDS-related cause” refer to deaths adjudicated as non–AIDS-related or AIDS-related, respectively, but without sufficient information to assign a more specific underlying cause category. “Undetermined mortality cause” refers to deaths for which AIDS-related versus non–AIDS-related status could not be determined.

***Supplementary Table 7. Relationship between numbers and combinations of metabolic conditions, modelled as time-varying exposure, and all-cause (top part) and non-AIDS related (bottom part) mortality***

| **Group of metabolic comorbidity** | **Mortality** | **PY** | **Mortality rate per 1000 PY (95% CI)** | **Adjusted HR (95% CI)** | ***p* value** |
| --- | --- | --- | --- | --- | --- |
| **All-cause mortality** |  |  |  |  |  |
| **Number of conditions** |  |  |  |  |  |
| 0 | 197 | 27 446·4 | 7·2 (6·2–8·3) | Reference | – |
| 1 | 1 327 | 129 169·0 | 10·3 (9·7–10·8) | 1·7 (1·5–2·0) | < 0·001 |
| ≥2 | 488 | 30 664·1 | 15·9 (14·5–17·4) | 2·1 (1·8–2·5) | < 0·001 |
| **Combinations** |  |  |  |  |  |
| No comorbidity | 197 | 27 446·4 | 7·2 (6·2–8·3) | Reference | – |
| Single condition | 1 327 | 129 169·0 | 10·3 (9·7–10·8) | 1·7 (1·5–2·0) | < 0·001 |
| Hypertension–diabetes–dyslipidaemia doublet | 345 | 19 116·2 | 18·1 (16·2–20·1) | 2·1 (1·8–2·6) | < 0·001 |
| Hypertension–diabetes–dyslipidaemia triad | 99 | 2 302·6 | 43·0 (34·9–52·4) | 4·1 (3·2–5·3) | < 0·001 |
| Other MM combinations | 44 | 9 245·3 | 4·8 (3·5–6·4) | 1·0 (0·7–1·4) | 0·960 |
| **Non–AIDS-related mortality** |  |  |  |  |  |
| **Number of conditions** |  |  |  |  |  |
| 0 | 138 | 27 446·4 | 5·0 (4·2–5·9) | Reference | – |
| 1 | 941 | 129 169·0 | 7·3 (6·8–7·8) | 1·8 (1·5–2·2) | < 0·001 |
| ≥2 | 372 | 30 664·1 | 12·1 (10·9–13·4) | 2·2 (1·8–2·7) | < 0·001 |
| **Combinations** |  |  |  |  |  |
| No comorbidity | 138 | 27 446·4 | 5·0 (4·2–5·9) | Reference | – |
| Single condition | 941 | 129 169·0 | 7·3 (6·8–7·8) | 1·8 (1·5–2·1) | < 0·001 |
| Hypertension–diabetes–dyslipidaemia doublet | 267 | 19 116·2 | 14·0 (12·3–15·8) | 2·3 (1·8–2·8) | < 0·001 |
| Hypertension–diabetes–dyslipidaemia triad | 76 | 2 302·6 | 33·0 (26·0–41·3) | 4·2 (3·2–5·7) | < 0·001 |
| Other MM combinations | 29 | 9 245·3 | 3·1 (2·1–4·5) | 0·9 (0·6–1·4) | 0·653 |

MM, metabolic multimorbidity; PWH, people with HIV; PY, person-years; hypertension–diabetes–dyslipidaemia doublet, any two of hypertension, diabetes, and dyslipidaemia; hypertension–diabetes–dyslipidaemia triad, coexistence of hypertension, diabetes, and dyslipidaemia. Mortality rates (per 1000 person-years) and adjusted hazard ratios (aHRs) with 95% confidence intervals (CIs) were estimated using Cox regression models with time-dependent MM. One model used metabolic comorbidity counts (0, 1, and ≥2 conditions), and the other used MM combinations (no comorbidity, single condition, hypertension–diabetes–dyslipidaemia doublet, hypertension–diabetes–dyslipidaemia triad, and other MM combinations) as the primary exposure, with the no comorbidity group as the reference, while adjusting for covariates.

***Supplementary Table 8. Relationship between numbers of metabolic conditions, modelled as time-varying exposure, and metabolic-related mortality***

| **Variable** | **Adjusted HR** | **95% CI** | ***p* value** |
| --- | --- | --- | --- |
| **Number of conditions** |  |  |  |
| 0 (Reference) | 1·0 | – | – |
| 1 | 1·4 | 1·0–2·1 | 0·075 |
| ≥2 | 2·5 | 1·7–3·8 | < 0·001 |
| **Age at ART initiation (years)** |  |  |  |
| 18–25 (Reference) | 1·0 | – | – |
| 26–35 | 1·4 | 0·5–3·8 | 0·470 |
| 36–45 | 2·2 | 0·8–5·9 | 0·115 |
| >45 | 11·2 | 4·3–29·1 | < 0·001 |
| **Sex** |  |  |  |
| Female (Reference) | 1·0 | – | – |
| Male | 1·5 | 1·1–2·0 | 0·020 |
| **Baseline CD4+ T-cell (cells/µL)** |  |  |  |
| ≤200 (Reference) | 1·0 | – | – |
| 201–350 | 1·1 | 0·8–1·4 | 0·638 |
| 351–500 | 0·9 | 0·6–1·4 | 0·696 |
| >500 | 0·9 | 0·6–1·6 | 0·828 |
| **Marital status** |  |  |  |
| Unmarried (Reference) | 1·0 | – | – |
| Married | 0·9 | 0·6–1·4 | 0·679 |
| Divorced or widowed | 1·2 | 0·7–1·9 | 0·522 |
| Others | 0·9 | 0·2–3·9 | 0·906 |
| **Route of HIV transmission** |  |  |  |
| MSM (Reference) | 1·0 | – | – |
| IDU | 4·5 | 1·9–10·9 | 0·003 |
| Heterosexual | 4·4 | 2·8–7·0 | < 0·001 |
| Others | 7·3 | 3·3–16·4 | < 0·001 |
| **Initial ART regimen** |  |  |  |
| INSTI-based (Reference) | 1·0 | – | – |
| NNRTI-based | 1·7 | 0·9–2·9 | 0·084 |
| PI/r-based | 2·8 | 1·5–5·1 | 0·001 |
| Others | 3·9 | 1·1–13·6 | 0·039 |
| **HBV co-infection** |  |  |  |
| No (Reference) | 1·0 | – | – |
| Yes | 1·0 | 0·7–1·4 | 0·932 |
| **HCV co-infection** |  |  |  |
| No (Reference) | 1·0 | – | – |
| Yes | 1·1 | 0·6–1·9 | 0·848 |
| **Baseline HIV RNA (copies/mL)** |  |  |  |
| <5000 (Reference) | 1·0 | – | – |
| 5000–9999 | 0·8 | 0·4–1·6 | 0·614 |
| ≥10000 | 0·9 | 0·6–1·3 | 0·517 |
| WBC (×10⁹/L) | 1·1 | 1·0–1·2 | 0·105 |
| Platelet (×10⁹/L) | 1·0 | 0·9–1·1 | 0·705 |
| Creatinine (µmol/L) | 1·1 | 1·0–1·2 | 0·174 |
| FPG (mmol/L) | 1·1 | 1·0–1·2 | 0·008 |
| ALT (U/L) | 0·8 | 0·7–0·9 | 0·007 |
| AST (U/L) | 1·1 | 1·0–1·4 | 0·101 |
| HDL-C (mmol/L) | 0·8 | 0·7–0·9 | 0·006 |
| LDL-C (mmol/L) | 1·0 | 0·9–1·1 | 0·626 |
| TC (mmol/L) | 1·1 | 1·0–1·3 | 0·150 |
| TG (mmol/L) | 0·9 | 0·8–1·0 | 0·204 |

MM, metabolic multimorbidity; PWH, people with HIV; CD4, cluster of differentiation 4; IDU, injection drug use; MSM, men who have sex with men; ART, antiretroviral therapy; INSTI, integrase strand transfer inhibitor; NNRTI, non-nucleoside reverse transcriptase inhibitor; PI/r, ritonavir-boosted protease inhibitor; HBV, hepatitis B virus; HCV, hepatitis C virus; HIV RNA, human immunodeficiency virus ribonucleic acid; WBC, white blood cell; FPG, fasting plasma glucose; ALT, alanine aminotransferase; AST, aspartate aminotransferase; HDL-C, high-density lipoprotein cholesterol; LDL-C, low-density lipoprotein cholesterol; TC, total cholesterol; TG, triglycerides. Adjusted hazard ratios (aHRs) and 95% confidence intervals (CIs) were derived from Cox models. Number of conditions variable was the main exposure, reference categories are shown in parentheses.

***Supplementary Table 9. Relationship between combinations of metabolic conditions, modelled as time-varying exposure, and metabolic-related mortality***

| **Variable** | **Adjusted HR** | **95% CI** | ***p* value** |
| --- | --- | --- | --- |
| **Combinations** |  |  |  |
| No comorbidity (Reference) | 1·0 | – | – |
| Single condition | 1·4 | 1·0–2·1 | 0·085 |
| Hypertension–diabetes–dyslipidaemia doublet | 2·6 | 1·7–4·0 | < 0·001 |
| Hypertension–diabetes–dyslipidaemia triad | 5·1 | 3·0–8·7 | < 0·001 |
| Other MM combinations | 0·6 | 0·2–1·6 | 0·337 |
| **Age at ART initiation (years)** |  |  |  |
| 18–25 (Reference) | 1·0 | – | – |
| 26–35 | 1·5 | 0·6–3·9 | 0·437 |
| 36–45 | 2·3 | 0·9–6·1 | 0·102 |
| >45 | 11·3 | 4·3–29·3 | < 0·001 |
| **Sex** |  |  |  |
| Female (Reference) | 1·0 | – | – |
| Male | 1·5 | 1·1–2·0 | 0·022 |
| **Baseline CD4+ T-cell (cells/µL)** |  |  |  |
| ≤200 (Reference) | 1·0 | – | – |
| 201–350 | 1·1 | 0·8–1·4 | 0·683 |
| 351–500 | 0·9 | 0·6–1·3 | 0·663 |
| >500 | 0·9 | 0·6–1·6 | 0·790 |
| **Marital status** |  |  |  |
| Unmarried (Reference) | 1·0 | – | – |
| Married | 0·9 | 0·6–1·4 | 0·659 |
| Divorced or widowed | 1·1 | 0·7–1·8 | 0·568 |
| Others | 0·9 | 0·2–3·9 | 0·909 |
| **Route of HIV transmission** |  |  |  |
| MSM (Reference) | 1·0 | – | – |
| IDU | 4·4 | 1·8–10·7 | 0·002 |
| Heterosexual | 4·3 | 2·7–6·9 | < 0·001 |
| Others | 7·0 | 3·1–15·7 | < 0·001 |
| **Initial ART regimen** |  |  |  |
| INSTI-based (Reference) | 1·0 | – | – |
| NNRTI-based | 1·7 | 1·0–3·1 | 0·061 |
| PI/r-based | 2·9 | 1·6–5·4 | < 0·001 |
| Others | 4·0 | 1·1–14·1 | 0·036 |
| **HBV co-infection** |  |  |  |
| No (Reference) | 1·0 | – | – |
| Yes | 1·0 | 0·7–1·4 | 0·939 |
| **HCV co-infection** |  |  |  |
| No (Reference) | 1·0 | – | – |
| Yes | 1·1 | 0·6–1·9 | 0·850 |
| **Baseline HIV RNA (copies/mL)** |  |  |  |
| <5000 (Reference) | 1·0 | – | – |
| 5000–9999 | 0·9 | 0·4–1·7 | 0·648 |
| ≥10000 | 0·9 | 0·6–1·3 | 0·584 |
| WBC (×10⁹/L) | 1·1 | 1·0–1·2 | 0·122 |
| Platelet (×10⁹/L) | 1·0 | 0·9–1·1 | 0·736 |
| Creatinine (µmol/L) | 1·1 | 1·0–1·2 | 0·293 |
| FPG (mmol/L) | 1·1 | 1·0–1·2 | 0·085 |
| ALT (U/L) | 0·8 | 0·7–0·9 | 0·007 |
| AST (U/L) | 1·2 | 1·0–1·4 | 0·081 |
| HDL-C (mmol/L) | 0·8 | 0·7–0·9 | 0·006 |
| LDL-C (mmol/L) | 1·0 | 0·9–1·1 | 0·723 |
| TC (mmol/L) | 1·1 | 1·0–1·3 | 0·142 |
| TG (mmol/L) | 0·9 | 0·8–1·0 | 0·160 |

MM, metabolic multimorbidity; PWH, people with HIV; CD4, cluster of differentiation 4; IDU, injection drug use; MSM, men who have sex with men; ART, antiretroviral therapy; INSTI, integrase strand transfer inhibitor; NNRTI, non-nucleoside reverse transcriptase inhibitor; PI/r, ritonavir-boosted protease inhibitor; HBV, hepatitis B virus; HCV, hepatitis C virus; HIV RNA, human immunodeficiency virus ribonucleic acid; WBC, white blood cell; FPG, fasting plasma glucose; ALT, alanine aminotransferase; AST, aspartate aminotransferase; HDL-C, high-density lipoprotein cholesterol; LDL-C, low-density lipoprotein cholesterol; TC, total cholesterol; TG, triglycerides. Adjusted hazard ratios (aHRs) and 95% confidence intervals (CIs) were derived from Cox models. Combinations variable was the main exposure.

***Supplementary Table 10. Relationship between numbers of metabolic conditions, modelled as time-varying exposure, and all-cause mortality, by age at time of ART initiation***

| **Group of metabolic comorbidity** | **Mortality** | **PY** | **Mortality rate per 1000 PY (95% CI)** | **Adjusted HR (95% CI)** | ***p* value** |
| --- | --- | --- | --- | --- | --- |
| **Age at ART initiation ≤45 years** |  |  |  |  |  |
| **Number of conditions** |  |  |  |  |  |
| 0 | 84 | 20 688·3 | 4·1 (3·2–5·0) | Reference | – |
| 1 | 437 | 102 239·9 | 4·3 (3·9–4·7) | 1·1 (0·9–1·4) | 0·396 |
| ≥2 | 66 | 18 706·8 | 3·5 (2·7–4·5) | 1·1 (0·8–1·5) | 0·714 |
| **Combinations** |  |  |  |  |  |
| No comorbidity | 84 | 20 688·3 | 4·1 (3·2–5·0) | Reference | – |
| Single condition | 437 | 102 239·9 | 4·3 (3·9–4·7) | 1·1 (0·9–1·4) | 0·400 |
| Hypertension–diabetes–dyslipidaemia doublet | 48 | 11 067·5 | 4·3 (3·2–5·8) | 1·2 (0·8–1·8) | 0·343 |
| Hypertension–diabetes–dyslipidaemia triad | 9 | 803·7 | 11·2 (5·1–21·3) | 2·8 (1·4–5·8) | 0·010 |
| Other MM combinations | 9 | 6 835·7 | 1·3 (0·6–2·5) | 0·5 (0·2–1·0) | 0·059 |
| **Age at ART initiation >45 years** |  |  |  |  |  |
| **Number of conditions** |  |  |  |  |  |
| 0 | 113 | 6 758·1 | 16·7 (13·8–20·1) | Reference | – |
| 1 | 890 | 26 929·1 | 33·0 (30·9–35·3) | 2·4 (1·9–2·9) | < 0·001 |
| ≥2 | 422 | 11 957·2 | 35·3 (32·0–38·9) | 2·9 (2·3–3·5) | < 0·001 |
| **Combinations** |  |  |  |  |  |
| No comorbidity | 113 | 6 758·1 | 16·7 (13·8–20·1) | Reference | – |
| Single condition | 890 | 26 929·1 | 33·0 (30·9–35·3) | 2·3 (1·9–2·9) | < 0·001 |
| Hypertension–diabetes–dyslipidaemia doublet | 297 | 8 048·7 | 36·9 (32·8–41·3) | 2·9 (2·3–3·6) | < 0·001 |
| Hypertension–diabetes–dyslipidaemia triad | 90 | 1 498·9 | 60·0 (48·3–73·8) | 5·1 (3·8–6·8) | < 0·001 |
| Other MM combinations | 35 | 2 409·7 | 14·5 (10·1–20·2) | 1·5 (1·0–2·1) | 0·052 |

MM, metabolic multimorbidity; PWH, people with HIV; PY, person-years; “Hypertension–diabetes–dyslipidaemia doublet” refers to any two-condition combination among hypertension, diabetes, and dyslipidaemia. “Hypertension–diabetes–dyslipidaemia triad” refers to the coexistence of hypertension, diabetes, and dyslipidaemia. Mortality rates (per 1000 person-years) and adjusted hazard ratios (aHRs) with 95% confidence intervals (CIs) were estimated using Cox regression models with time-dependent MM. One model used metabolic comorbidity counts (0, 1, and ≥2 conditions), and the other used MM combination (no comorbidity, single condition, hypertension–diabetes–dyslipidaemia doublet, hypertension–diabetes–dyslipidaemia triad, and other MM combinations) as the primary exposure, with the no comorbidity group as the reference, while adjusting for covariates.

***Supplementary Table 11. Adjusted subdistribution hazard ratios of incident CKD and CVD in relation to the numbers and combinations of metabolic comorbidities***

| **Group of metabolic comorbidity** | **Events** | **PY** | **Incidence rate per 1000 PY (95% CI)** | **Adjusted sHR (95% CI)** | ***p* value** |
| --- | --- | --- | --- | --- | --- |
| **CKD** |  |  |  |  |  |
| **Number of conditions** |  |  |  |  |  |
| 0 | 17 | 27422·7 | 0·6 (0·4–1·0) | Reference | – |
| 1 | 195 | 128534·7 | 1·5 (1·3–1·7) | 2·4 (1·5–4·0) | < 0·001 |
| ≥2 | 170 | 29912·3 | 5·7 (4·9–6·6) | 6·2 (3·7–10·4) | < 0·001 |
| **Combinations** |  |  |  |  |  |
| No comorbidity | 17 | 27422·7 | 0·6 (0·4–1·0) | Reference | – |
| Single condition | 195 | 128534·7 | 1·5 (1·3–1·7) | 2·4 (1·5–4·0) | < 0·001 |
| Hypertension–diabetes–dyslipidaemia doublet | 98 | 18663·5 | 5·3 (4·3–6·4) | 5·9 (3·5–10·1) | < 0·001 |
| Hypertension–diabetes–dyslipidaemia triad | 37 | 2129·2 | 17·4 (12·2–24) | 15·5 (8·4–28·6) | < 0·001 |
| Other MM combinations | 35 | 9119·6 | 3·8 (2·7–5·3) | 4·8 (2·7–8·8) | < 0·001 |
| **CVD** |  |  |  |  |  |
| **Number of conditions** |  |  |  |  |  |
| 0 | 168 | 27397·3 | 6·1 (5·2–7·1) | Reference | – |
| 1 | 379 | 128367·7 | 3·0 (2·7–3·3) | 0·5 (0·4–0·6) | < 0·001 |
| ≥2 | 377 | 29697·5 | 12·7 (11·4–14) | 1·2 (1–1·5) | 0·036 |
| **Combinations** |  |  |  |  |  |
| No comorbidity | 168 | 27397·3 | 6·1 (5·2–7·1) | Reference | – |
| Single condition | 379 | 128367·7 | 3·0 (2·7–3·3) | 0·5 (0·4–0·6) | < 0·001 |
| Hypertension–diabetes–dyslipidaemia doublet | 263 | 18509·0 | 14·2 (12·5–16·0) | 1·3 (1·0–1·5) | 0·031 |
| Hypertension–diabetes–dyslipidaemia triad | 74 | 2034·0 | 36·4 (28·6–45·7) | 2·1 (1·6–2·8) | < 0·001 |
| Other MM combinations | 40 | 9154·6 | 4·4 (3·1–5·9) | 0·7 (0·5–1·0) | 0·070 |

MM, metabolic multimorbidity; PWH, people with HIV; PY, person-years; CKD, chronic kidney disease; CVD, cardiovascular disease. “Hypertension–diabetes–dyslipidaemia doublet” refers to any two-condition combination among hypertension, diabetes, and dyslipidaemia. “Hypertension–diabetes–dyslipidaemia triad” refers to the coexistence of hypertension, diabetes, and dyslipidaemia. Incidence rates (per 1000 person-years) were calculated for CKD and CVD. Adjusted subdistribution hazard ratios (sHRs) with 95% confidence intervals (CIs) were estimated using separate Fine–Gray competing-risk regression models for CKD and CVD, with all-cause death treated as a competing event. One model used metabolic comorbidity counts (0, 1, and ≥2 conditions), and the other used MM combinations (no comorbidity, single condition, hypertension–diabetes–dyslipidaemia doublet, hypertension–diabetes–dyslipidaemia triad, and other MM combinations) as the primary exposure, with the no comorbidity group as the reference, while adjusting for covariates.

***Supplementary Table 12. Tests of the proportional hazards assumption for covariates in the Cox regression model with MM parameterised by number of conditions***

| **Variable** | **Chi-square** | **df** | ***p* value** |
| --- | --- | --- | --- |
| Number of conditions | 22·2 | 2 | < 0·001 |
| Age at ART initiation | 13·0 | 3 | 0·005 |
| Sex | 0·5 | 1 | 0·498 |
| Baseline CD4+ T-cell | 23·3 | 3 | < 0·001 |
| Marital status | 8·4 | 3 | 0·039 |
| Route of HIV transmission | 3·4 | 3 | 0·338 |
| Initial ART regimen | 8·6 | 3 | 0·036 |
| HBV co-infection | 2·2 | 1 | 0·136 |
| HCV co-infection | 2·1 | 1 | 0·151 |
| Baseline HIV RNA | 1·9 | 2 | 0·392 |
| White blood cell count | 0·9 | 1 | 0·337 |
| Platelet count | 1·5 | 1 | 0·219 |
| Creatinine | 6·0 | 1 | 0·014 |
| Fasting plasma glucose | 3·6 | 1 | 0·060 |
| Alanine aminotransferase | 0·2 | 1 | 0·666 |
| Aspartate aminotransferase | 7·2 | 1 | 0·007 |
| High-density lipoprotein cholesterol | 13·6 | 1 | < 0·001 |
| Low-density lipoprotein cholesterol | 7·8 | 1 | 0·005 |
| Total cholesterol | 19·8 | 1 | < 0·001 |
| Triglycerides | 0·8 | 1 | 0·373 |
| Global test | 100·6 | 32 | < 0·001 |

MM, metabolic multimorbidity; ART, antiretroviral therapy; HBV, hepatitis B virus; HCV, hepatitis C virus; df, degrees of freedom. Proportional hazards assumptions were assessed for each covariate and globally using tests based on scaled Schoenfeld residuals. A two-sided *p* value < 0·05 was considered evidence against the proportional hazards assumption. The global test evaluates whether the Cox model as a whole satisfies the proportional hazards assumption.

***Supplementary Table 13. Tests of the proportional hazards assumption for covariates in the Cox regression model with MM parameterised by combinations***

| **Variable** | **Chi-square** | **df** | ***p* value** |
| --- | --- | --- | --- |
| Combinations | 25·7 | 4 | < 0·001 |
| Age at ART initiation | 12·3 | 3 | 0·006 |
| Sex | 0·7 | 1 | 0·418 |
| Baseline CD4+ T-cell | 24·0 | 3 | < 0·001 |
| Marital status | 7·9 | 3 | 0·047 |
| Route of HIV transmission | 3·2 | 3 | 0·358 |
| Initial ART regimen | 8·3 | 3 | 0·040 |
| HBV co-infection | 2·1 | 1 | 0·146 |
| HCV co-infection | 2·3 | 1 | 0·132 |
| Baseline HIV RNA | 2·0 | 2 | 0·366 |
| White blood cell count | 0·8 | 1 | 0·359 |
| Platelet count | 1·6 | 1 | 0·212 |
| Creatinine | 5·5 | 1 | 0·019 |
| Fasting plasma glucose | 3·2 | 1 | 0·076 |
| Alanine aminotransferase | 0·3 | 1 | 0·570 |
| Aspartate aminotransferase | 7·0 | 1 | 0·008 |
| High-density lipoprotein cholesterol | 13·1 | 1 | < 0·001 |
| Low-density lipoprotein cholesterol | 7·7 | 1 | 0·006 |
| Total cholesterol | 19·5 | 1 | < 0·001 |
| Triglycerides | 0·6 | 1 | 0·447 |
| Global test | 100·0 | 34 | < 0·001 |

MM, metabolic multimorbidity; ART, antiretroviral therapy; HBV, hepatitis B virus; HCV, hepatitis C virus; df, degrees of freedom. Proportional hazards assumptions were assessed for each covariate and globally using tests based on scaled Schoenfeld residuals. A two-sided *p* value < 0·05 was considered evidence against the proportional hazards assumption. The global test evaluates whether the Cox model as a whole satisfies the proportional hazards assumption.

***Supplementary Table 14. Time-stratified adjusted hazard ratios for all-cause mortality and non–AIDS-related mortality according to number of conditions and MM combinations***

1. Number of conditions

| **Outcome** | **Follow-up period** | **Group** | **Adjusted HR (95% CI)** | ***p* value** |
| --- | --- | --- | --- | --- |
| All-cause mortality | 0–1 year | 0 conditions | Reference | – |
|  |  | 1 condition | 2·7 (1·9–4·0) | < 0·001 |
|  |  | ≥2 conditions | 2·3 (1·4–3·6) | < 0·001 |
|  | 1–5 years | 0 conditions | Reference | – |
|  |  | 1 condition | 1·8 (1·5–2·2) | < 0·001 |
|  |  | ≥2 conditions | 2·1 (1·7–2·7) | < 0·001 |
|  | >5 years | 0 conditions | Reference | – |
|  |  | 1 condition | 1·2 (0·9–1·6) | 0·386 |
|  |  | ≥2 conditions | 1·6 (1·2–2·2) | 0·003 |
| Non–AIDS-related mortality | 0–1 year | 0 conditions | Reference | – |
|  |  | 1 condition | 3·9 (2·3–6·8) | < 0·001 |
|  |  | ≥2 conditions | 3·2 (1·7–6·1) | < 0·001 |
|  | 1–5 years | 0 conditions | Reference | – |
|  |  | 1 condition | 1·8 (1·4–2·2) | < 0·001 |
|  |  | ≥2 conditions | 2·3 (1·7–3·0) | < 0·001 |
|  | >5 years | 0 conditions | Reference | – |
|  |  | 1 condition | 1·2 (0·8–1·7) | 0·340 |
|  |  | ≥2 conditions | 1·5 (1·1–2·2) | 0·018 |

1. Combinations

| **Outcome** | **Follow-up period** | **Group** | **Adjusted HR (95% CI)** | ***p* value** |
| --- | --- | --- | --- | --- |
| All-cause mortality | 0–1 year | No comorbidity | Reference | – |
|  |  | Single condition | 2·7 (1·9–4·0) | < 0·001 |
|  |  | Hypertension–diabetes–dyslipidaemia doublet | 2·7 (1·7–4·4) | < 0·001 |
|  |  | Hypertension–diabetes–dyslipidaemia triad | 2·9 (1·1–7·5) | 0·031 |
|  |  | Other MM combinations | 0·8 (0·2–2·5) | 0·637 |
|  | 1–5 years | No comorbidity | Reference | – |
|  |  | Single condition | 1·8 (1·5–2·2) | < 0·001 |
|  |  | Hypertension–diabetes–dyslipidaemia doublet | 2·3 (1·8–2·9) | < 0·001 |
|  |  | Hypertension–diabetes–dyslipidaemia triad | 3·7 (2·6–5·3) | < 0·001 |
|  |  | Other MM combinations | 1·1 (0·7–1·7) | 0·787 |
|  | >5 years | No comorbidity | Reference | – |
|  |  | Single condition | 1·1 (0·9–1·5) | 0·386 |
|  |  | Hypertension–diabetes–dyslipidaemia doublet | 1·5 (1·1–2·1) | 0·015 |
|  |  | Hypertension–diabetes–dyslipidaemia triad | 3·8 (2·5–5·6) | < 0·001 |
|  |  | Other MM combinations | 0·8 (0·4–1·3) | 0·337 |
| Non–AIDS-related mortality | 0–1 year | No comorbidity | Reference | – |
|  |  | Single condition | 3·9 (2·3–6·8) | < 0·001 |
|  |  | Hypertension–diabetes–dyslipidaemia doublet | 3·9 (2·0–7·5) | < 0·001 |
|  |  | Hypertension–diabetes–dyslipidaemia triad | 5·0 (1·6–15·5) | 0·005 |
|  |  | Other MM combinations | 0·6 (0·1–4·2) | 0·570 |
|  | 1–5 years | No comorbidity | Reference | – |
|  |  | Single condition | 1·8 (1·4–2·2) | < 0·001 |
|  |  | Hypertension–diabetes–dyslipidaemia doublet | 2·5 (1·9–3·3) | < 0·001 |
|  |  | Hypertension–diabetes–dyslipidaemia triad | 3·8 (2·5–5·8) | < 0·001 |
|  |  | Other MM combinations | 1·0 (0·6–1·6) | 0·849 |
|  | >5 years | No comorbidity | Reference | – |
|  |  | Single condition | 1·2 (0·8–1·7) | 0·365 |
|  |  | Hypertension–diabetes–dyslipidaemia doublet | 1·4 (1·0–2·1) | 0·059 |
|  |  | Hypertension–diabetes–dyslipidaemia triad | 3·6 (2·3–5·6) | < 0·001 |
|  |  | Other MM combinations | 0·7 (0·4–1·4) | 0·298 |

HR, hazard ratio; CI, confidence interval; MM, metabolic multimorbidity; ART, antiretroviral therapy; HBV, hepatitis B virus; HCV, hepatitis C virus. Adjusted HRs and 95% CIs were estimated using time-stratified Cox proportional hazards models for all-cause mortality and non–AIDS-related mortality. Follow-up was divided into 0–1 year, 1–5 years, and >5 years according to the time-varying pattern of associations identified in the proportional hazards assessment. In the count-based model, metabolic comorbidity burden was classified as 0, 1, and ≥2 conditions. In the phenotype-based model, MM combinations were classified as no comorbidity, single, hypertension–diabetes–dyslipidaemia doublet, and hypertension–diabetes–dyslipidaemia triad. “Hypertension–diabetes–dyslipidaemia doublet” refers to any two-condition combination among hypertension, diabetes, and dyslipidaemia, and “hypertension–diabetes–dyslipidaemia triad” refers to the coexistence of all three conditions. The reference group was 0 conditions in the count-based model and no comorbidity in the phenotype-based model. Models were adjusted for age at ART initiation, sex, baseline CD4+ T-cell count, marital status, route of HIV transmission, initial ART regimen, HBV co-infection, HCV co-infection, baseline HIV RNA, white blood cell count, platelet count, creatinine, fasting plasma glucose, alanine aminotransferase, aspartate aminotransferase, high-density lipoprotein cholesterol, low-density lipoprotein cholesterol, total cholesterol, and triglycerides. *p* values were two-sided.

***Supplementary Table 15. Relationship between numbers of metabolic conditions, modelled as time-varying exposure, and other covariates with all-cause mortality***

| **Variable** | **Adjusted HR** | **95% CI** | ***p* value** |
| --- | --- | --- | --- |
| **Number of conditions** |  |  |  |
| 0 (Reference) | 1·0 | – | – |
| 1 | 1·8 | 1·5–2·0 | < 0·001 |
| ≥2 | 2·1 | 1·8–2·5 | < 0·001 |
| **Age at ART initiation (years)** |  |  |  |
| 18–25 (Reference) | 1·0 | – | – |
| 26–35 | 1·2 | 0·9–1·6 | 0·353 |
| 36–45 | 2·0 | 1·5–2·7 | < 0·001 |
| >45 | 6·3 | 4·7–8·5 | < 0·001 |
| **Sex** |  |  |  |
| Female (Reference) | 1·0 | – | – |
| Male | 1·7 | 1·5–2·0 | < 0·001 |
| **Baseline CD4+ T-cell (cells/µL)** |  |  |  |
| ≤200 (Reference) | 1·0 | – | – |
| 201–350 | 0·9 | 0·8–1·0 | 0·014 |
| 351–500 | 0·8 | 0·7–0·9 | 0·005 |
| >500 | 0·7 | 0·5–0·9 | 0·001 |
| **Marital status** |  |  |  |
| Unmarried (Reference) | 1·0 | – | – |
| Married | 0·8 | 0·7–0·9 | 0·002 |
| Divorced or widowed | 1·1 | 0·9–1·3 | 0·341 |
| Others | 1·1 | 0·7–1·8 | 0·745 |
| **Route of HIV transmission** |  |  |  |
| MSM (Reference) | 1·0 | – | – |
| IDU | 6·2 | 4·7–8·1 | < 0·001 |
| Heterosexual | 4·0 | 3·4–4·8 | < 0·001 |
| Others | 5·3 | 3·7–7·6 | < 0·001 |
| **Initial ART regimen** |  |  |  |
| INSTI-based (Reference) | 1·0 | – | – |
| NNRTI-based | 1·5 | 1·2–1·9 | < 0·001 |
| PI/r-based | 2·6 | 2·0–3·2 | < 0·001 |
| Others | 2·9 | 1·7–4·9 | < 0·001 |
| **HBV co-infection** |  |  |  |
| No (Reference) | 1·0 | – | – |
| Yes | 1·1 | 1·0–1·3 | 0·113 |
| **HCV co-infection** |  |  |  |
| No (Reference) | 1·0 | – | – |
| Yes | 1·5 | 1·2–1·8 | < 0·001 |
| **Baseline HIV RNA (copies/mL)** |  |  |  |
| <5000 (Reference) | 1·0 | – | – |
| 5000–9999 | 0·9 | 0·7–1·1 | 0·278 |
| ≥10000 | 0·9 | 0·8–1·1 | 0·241 |
| WBC (×10⁹/L) | 1·1 | 1·0–1·1 | < 0·001 |
| Platelet (×10⁹/L) | 1·0 | 1·0–1·0 | 0·853 |
| Creatinine (µmol/L) | 1·0 | 1·0–1·1 | 0·466 |
| FPG (mmol/L) | 1·1 | 1·0–1·1 | < 0·001 |
| ALT (U/L) | 0·7 | 0·7–0·8 | < 0·001 |
| AST (U/L) | 1·3 | 1·2–1·4 | < 0·001 |
| HDL-C (mmol/L) | 0·8 | 0·8–0·8 | < 0·001 |
| LDL-C (mmol/L) | 1·0 | 1·0–1·1 | 0·793 |
| TC (mmol/L) | 1·0 | 1·0–1·1 | 0·551 |
| TG (mmol/L) | 0·9 | 0·9–1·0 | 0·001 |

MM, metabolic multimorbidity; PWH, people with HIV; CD4, cluster of differentiation 4; IDU, injection drug use; MSM, men who have sex with men; ART, antiretroviral therapy; INSTI, integrase strand transfer inhibitor; NNRTI, non-nucleoside reverse transcriptase inhibitor; PI/r, ritonavir-boosted protease inhibitor; HBV, hepatitis B virus; HCV, hepatitis C virus; HIV RNA, human immunodeficiency virus ribonucleic acid; WBC, white blood cell; FPG, fasting plasma glucose; ALT, alanine aminotransferase; AST, aspartate aminotransferase; HDL-C, high-density lipoprotein cholesterol; LDL-C, low-density lipoprotein cholesterol; TC, total cholesterol; TG, triglycerides. Adjusted hazard ratios (aHRs) and 95% confidence intervals (CIs) were derived from Cox models. Number of conditions (0, 1, ≥2 conditions) was the main exposure, reference categories are shown in parentheses.

***Supplementary Table 16. Relationship between combinations of metabolic conditions, modelled as time-varying exposure, and other covariates with all-cause mortality***

| **Variable** | **Adjusted HR** | **95% CI** | ***p* value** |
| --- | --- | --- | --- |
| **Combinations** |  |  |  |
| No comorbidity (Reference) | 1·0 | – | – |
| Single condition | 1·7 | 1·5–2·0 | < 0·001 |
| Hypertension–diabetes–dyslipidaemia doublet | 2·1 | 1·8–2·6 | < 0·001 |
| Hypertension–diabetes–dyslipidaemia triad | 4·1 | 3·2–5·3 | < 0·001 |
| Other MM combinations | 1·0 | 0·7–1·4 | 0·960 |
| **Age at ART initiation (years)** |  |  |  |
| 18–25 (Reference) | 1·0 | – | – |
| 26–35 | 1·2 | 0·9–1·6 | 0·309 |
| 36–45 | 2·0 | 1·5–2·8 | < 0·001 |
| >45 | 6·3 | 4·7–8·5 | < 0·001 |
| **Sex** |  |  |  |
| Female (Reference) | 1·0 | – | – |
| Male | 1·7 | 1·5–2·0 | < 0·001 |
| **Baseline CD4+ T-cell (cells/µL)** |  |  |  |
| ≤200 (Reference) | 1·0 | – | – |
| 201–350 | 0·9 | 0·8–1·0 | 0·010 |
| 351–500 | 0·8 | 0·7–0·9 | 0·004 |
| >500 | 0·7 | 0·5–0·9 | 0·001 |
| **Marital status** |  |  |  |
| Unmarried (Reference) | 1·0 | – | – |
| Married | 0·8 | 0·7–0·9 | 0·002 |
| Divorced or widowed | 1·1 | 0·9–1·3 | 0·394 |
| Others | 1·1 | 0·7–1·8 | 0·743 |
| **Route of HIV transmission** |  |  |  |
| MSM (Reference) | 1·0 | – | – |
| IDU | 6·2 | 4·7–8·1 | < 0·001 |
| Heterosexual | 4·0 | 3·3–4·7 | < 0·001 |
| Others | 5·2 | 3·6–7·4 | < 0·001 |
| **Initial ART regimen** |  |  |  |
| INSTI-based (Reference) | 1·0 | – | – |
| NNRTI-based | 1·6 | 1·3–2·0 | < 0·001 |
| PI/r-based | 2·6 | 2·1–3·3 | < 0·001 |
| Others | 2·9 | 1·7–4·9 | < 0·001 |
| **HBV** |  |  |  |
| No (Reference) | 1·0 | – | – |
| Yes | 1·1 | 1·0–1·3 | 0·109 |
| **HCV** |  |  |  |
| No (Reference) | 1·0 | – | – |
| Yes | 1·5 | 1·2–1·8 | < 0·001 |
| **Baseline HIV RNA (copies/mL)** |  |  |  |
| <5000 (Reference) | 1·0 | – | – |
| 5000–9999 | 0·9 | 0·7–1·1 | 0·299 |
| ≥10000 | 0·9 | 0·8–1·1 | 0·288 |
| WBC (×10⁹/L) | 1·1 | 1·0–1·1 | < 0·001 |
| Platelet (×10⁹/L) | 1·0 | 1·0–1·0 | 0·921 |
| Creatinine (µmol/L) | 1·0 | 1·0–1·1 | 0·730 |
| FPG (mmol/L) | 1·1 | 1·0–1·1 | 0·016 |
| ALT (U/L) | 0·7 | 0·7–0·8 | < 0·001 |
| AST (U/L) | 1·3 | 1·3–1·4 | < 0·001 |
| HDL-C (mmol/L) | 0·8 | 0·8–0·8 | < 0·001 |
| LDL-C (mmol/L) | 1·0 | 1·0–1·1 | 0·676 |
| TC (mmol/L) | 1·0 | 1·0–1·1 | 0·510 |
| TG (mmol/L) | 0·9 | 0·9–1·0 | < 0·001 |

MM, metabolic multimorbidity; PWH, people with HIV; CD4, cluster of differentiation 4; IDU, injection drug use; MSM, men who have sex with men; ART, antiretroviral therapy; INSTI, integrase strand transfer inhibitor; NNRTI, non-nucleoside reverse transcriptase inhibitor; PI/r, ritonavir-boosted protease inhibitor; HBV, hepatitis B virus; HCV, hepatitis C virus; HIV RNA, human immunodeficiency virus ribonucleic acid; WBC, white blood cell; FPG, fasting plasma glucose; ALT, alanine aminotransferase; AST, aspartate aminotransferase; HDL-C, high-density lipoprotein cholesterol; LDL-C, low-density lipoprotein cholesterol; TC, total cholesterol; TG, triglycerides; hypertension–diabetes–dyslipidaemia doublet, any two of hypertension, diabetes, and dyslipidaemia; hypertension–diabetes–dyslipidaemia triad, coexistence of hypertension, diabetes, and dyslipidaemia. Adjusted hazard ratios (aHRs) and 95% confidence intervals (CIs) were derived from Cox models. Combinations (No comorbidity, single condition, hypertension–diabetes–dyslipidaemia doublet, hypertension–diabetes–dyslipidaemia triad, other MM combinations) were the main exposure, reference categories are shown in parentheses.

***Supplementary Table 17. Relationship between numbers of metabolic conditions, modelled as time-varying exposure, and other covariates with non-AIDS-related mortality***

| **Variable** | **Adjusted HR** | **95% CI** | ***p* value** |
| --- | --- | --- | --- |
| **Number of conditions** |  |  |  |
| 0 (Reference) | 1·0 | – | – |
| 1 | 1·8 | 1·5–2·2 | < 0·001 |
| ≥2 | 2·2 | 1·8–2·7 | < 0·001 |
| **Age at ART initiation (years)** |  |  |  |
| 18–25 (Reference) | 1·0 | – | – |
| 26–35 | 1·1 | 0·8–1·6 | 0·647 |
| 36–45 | 2·1 | 1·5–3·0 | < 0·001 |
| >45 | 7·1 | 4·9–10·1 | < 0·001 |
| **Sex** |  |  |  |
| Female (Reference) | 1·0 | – | – |
| Male | 1·7 | 1·4–1·9 | < 0·001 |
| **Baseline CD4+ T-cell (cells/µL)** |  |  |  |
| ≤200 (Reference) | 1·0 | – | – |
| 201–350 | 0·9 | 0·8–1·1 | 0·230 |
| 351–500 | 0·9 | 0·7–1·0 | 0·142 |
| >500 | 0·8 | 0·6–1·0 | 0·080 |
| **Marital status** |  |  |  |
| Unmarried (Reference) | 1·0 | – | – |
| Married | 0·8 | 0·6–0·9 | 0·003 |
| Divorced or widowed | 1·1 | 0·9–1·3 | 0·621 |
| Others | 0·8 | 0·4–1·6 | 0·601 |
| **Route of HIV transmission** |  |  |  |
| MSM (Reference) | 1·0 | – | – |
| IDU | 5·8 | 4·1–8·1 | < 0·001 |
| Heterosexual | 4·1 | 3·3–5·1 | < 0·001 |
| Others | 5·8 | 3·8–8·7 | < 0·001 |
| **Initial ART regimen** |  |  |  |
| INSTI-based (Reference) | 1·0 | – | – |
| NNRTI-based | 1·6 | 1·2–2·1 | 0·001 |
| PI/r-based | 2·8 | 2·1–3·7 | < 0·001 |
| Others | 4·1 | 2·3–7·1 | < 0·001 |
| **HBV co-infection** |  |  |  |
| No (Reference) | 1·0 | – | – |
| Yes | 1·2 | 1·0–1·4 | 0·038 |
| **HCV co-infection** |  |  |  |
| No (Reference) | 1·0 | – | – |
| Yes | 1·5 | 1·2–1·9 | 0·001 |
| **Baseline HIV RNA (copies/mL)** |  |  |  |
| <5000 (Reference) | 1·0 | – | – |
| 5000–9999 | 0·8 | 0·6–1·1 | 0·241 |
| ≥10000 | 1·0 | 0·8–1·2 | 0·627 |
| WBC (×10⁹/L) | 1·1 | 1·1–1·2 | < 0·001 |
| Platelet (×10⁹/L) | 1·0 | 0·9–1·0 | 0·662 |
| Creatinine (µmol/L) | 1·0 | 1·0–1·1 | 0·152 |
| FPG (mmol/L) | 1·1 | 1·0–1·1 | 0·001 |
| ALT (U/L) | 0·7 | 0·7–0·8 | < 0·001 |
| AST (U/L) | 1·3 | 1·2–1·4 | < 0·001 |
| HDL-C (mmol/L) | 0·8 | 0·8–0·9 | < 0·001 |
| LDL-C (mmol/L) | 1·0 | 1·0–1·1 | 0·763 |
| TC (mmol/L) | 1·1 | 1·0–1·1 | 0·043 |
| TG (mmol/L) | 0·9 | 0·9–1·0 | < 0·001 |

MM, metabolic multimorbidity; PWH, people with HIV; CD4, cluster of differentiation 4; IDU, injection drug use; MSM, men who have sex with men; ART, antiretroviral therapy; INSTI, integrase strand transfer inhibitor; NNRTI, non-nucleoside reverse transcriptase inhibitor; PI/r, ritonavir-boosted protease inhibitor; HBV, hepatitis B virus; HCV, hepatitis C virus; HIV RNA, human immunodeficiency virus ribonucleic acid; WBC, white blood cell; FPG, fasting plasma glucose; ALT, alanine aminotransferase; AST, aspartate aminotransferase; HDL-C, high-density lipoprotein cholesterol; LDL-C, low-density lipoprotein cholesterol; TC, total cholesterol; TG, triglycerides. Adjusted hazard ratios (aHRs) and 95% confidence intervals (CIs) were derived from Cox models. Number of conditions (0, 1, ≥2 conditions) was the main exposure, reference categories are shown in parentheses.

***Supplementary Table 18. Relationship between combinations of metabolic conditions, modelled as time-varying exposure, and other covariates with non-AIDS-related mortality***

| **Variable** | **Adjusted HR** | **95% CI** | ***p* value** |
| --- | --- | --- | --- |
| **Combinations** |  |  |  |
| No comorbidity (Reference) | 1·0 | – | – |
| Single condition | 1·8 | 1·5–2·1 | < 0·001 |
| Hypertension–diabetes–dyslipidaemia doublet | 2·3 | 1·8–2·8 | < 0·001 |
| Hypertension–diabetes–dyslipidaemia triad | 4·2 | 3·2–5·7 | < 0·001 |
| Other MM combinations | 0·9 | 0·6–1·4 | 0·653 |
| **Age at ART initiation (years)** |  |  |  |
| 18–25 (Reference) | 1·0 | – | – |
| 26–35 | 1·1 | 0·8–1·6 | 0·591 |
| 36–45 | 2·1 | 1·5–3·1 | < 0·001 |
| >45 | 7·1 | 4·9–10·1 | < 0·001 |
| **Sex** |  |  |  |
| Female (Reference) | 1·0 | – | – |
| Male | 1·7 | 1·4–1·9 | < 0·001 |
| **Baseline CD4+ T-cell (cells/µL)** |  |  |  |
| ≤200 (Reference) | 1·0 | – | – |
| 201–350 | 0·9 | 0·8–1·0 | 0·196 |
| 351–500 | 0·9 | 0·7–1·0 | 0·131 |
| >500 | 0·8 | 0·6–1·0 | 0·073 |
| **Marital status** |  |  |  |
| Unmarried (Reference) | 1·0 | – | – |
| Married | 0·8 | 0·6–0·9 | 0·003 |
| Divorced or widowed | 1·0 | 0·9–1·3 | 0·696 |
| Others | 0·8 | 0·4–1·6 | 0·605 |
| **Route of HIV transmission** |  |  |  |
| MSM (Reference) | 1·0 | – | – |
| IDU | 5·7 | 4·1–8·0 | < 0·001 |
| Heterosexual | 4·1 | 3·3–5·0 | < 0·001 |
| Others | 5·6 | 3·7–8·4 | < 0·001 |
| **Initial ART regimen** |  |  |  |
| INSTI-based (Reference) | 1·0 | – | – |
| NNRTI-based | 1·6 | 1·3–2·2 | < 0·001 |
| PI/r-based | 2·8 | 2·1–3·8 | < 0·001 |
| Others | 4·1 | 2·3–7·2 | < 0·001 |
| **HBV co-infection** |  |  |  |
| No (Reference) | 1·0 | – | – |
| Yes | 1·2 | 1·0–1·4 | 0·037 |
| **HCV co-infection** |  |  |  |
| No (Reference) | 1·0 | – | – |
| Yes | 1·5 | 1·2–1·9 | 0·001 |
| **Baseline HIV RNA (copies/mL)** |  |  |  |
| <5000 (Reference) | 1·0 | – | – |
| 5000–9999 | 0·8 | 0·6–1·2 | 0·258 |
| ≥10000 | 1·0 | 0·8–1·2 | 0·703 |
| WBC (×10⁹/L) | 1·1 | 1·1–1·2 | < 0·001 |
| Platelet (×10⁹/L) | 1·0 | 0·9–1·0 | 0·717 |
| Creatinine (µmol/L) | 1·0 | 1·0–1·1 | 0·287 |
| FPG (mmol/L) | 1·1 | 1·0–1·1 | 0·038 |
| ALT (U/L) | 0·7 | 0·7–0·8 | < 0·001 |
| AST (U/L) | 1·3 | 1·2–1·4 | < 0·001 |
| HDL-C (mmol/L) | 0·8 | 0·8–0·9 | < 0·001 |
| LDL-C (mmol/L) | 1·0 | 1·0–1·1 | 0·653 |
| TC (mmol/L) | 1·1 | 1·0–1·2 | 0·037 |
| TG (mmol/L) | 0·9 | 0·9–1·0 | < 0·001 |

MM, metabolic multimorbidity; PWH, people with HIV; CD4, cluster of differentiation 4; IDU, injection drug use; MSM, men who have sex with men; ART, antiretroviral therapy; INSTI, integrase strand transfer inhibitor; NNRTI, non-nucleoside reverse transcriptase inhibitor; PI/r, ritonavir-boosted protease inhibitor; HBV, hepatitis B virus; HCV, hepatitis C virus; HIV RNA, human immunodeficiency virus ribonucleic acid; WBC, white blood cell; FPG, fasting plasma glucose; ALT, alanine aminotransferase; AST, aspartate aminotransferase; HDL-C, high-density lipoprotein cholesterol; LDL-C, low-density lipoprotein cholesterol; TC, total cholesterol; TG, triglycerides; hypertension–diabetes–dyslipidaemia doublet, any two of hypertension, diabetes, and dyslipidaemia; hypertension–diabetes–dyslipidaemia triad, coexistence of hypertension, diabetes, and dyslipidaemia. Adjusted hazard ratios (aHRs) and 95% confidence intervals (CIs) were derived from Cox models. Combinations variable (No comorbidity, single condition, hypertension–diabetes–dyslipidaemia doublet, hypertension–diabetes–dyslipidaemia triad, other MM combinations) was the main exposure, reference categories are shown in parentheses.

***Supplementary Table 19. Complete-case sensitivity analysis of the associations of the number of metabolic conditions and combinations with all-cause and non–AIDS-related mortality***

| **Group of metabolic comorbidity** | **Mortality** | **PY** | **Mortality rate per 1000 PY (95% CI)** | **Adjusted HR (95% CI)** | ***p* value** |
| --- | --- | --- | --- | --- | --- |
| **All-cause mortality** |  |  |  |  |  |
| **Number of conditions** |  |  |  |  |  |
| 0 | 197 | 27 446·4 | 7·2 (6·2–8·3) | Reference | – |
| 1 | 1 327 | 129 169·0 | 10·3 (9·7–10·8) | 1·6 (1·4–1·9) | < 0·001 |
| ≥2 | 488 | 30 664·1 | 15·9 (14·5–17·4) | 1·9 (1·5–2·3) | < 0·001 |
| **Combinations** |  |  |  |  |  |
| No comorbidity | 197 | 27 446·4 | 7·2 (6·2–8·3) | Reference | – |
| Single condition | 1 327 | 129 169·0 | 10·3 (9·7–10·8) | 1·6 (1·3–1·9) | < 0·001 |
| Hypertension–diabetes–dyslipidaemia doublet | 345 | 19 116·2 | 18·1 (16·2–20·1) | 1·9 (1·6–2·3) | < 0·001 |
| Hypertension–diabetes–dyslipidaemia triad | 99 | 2 302·6 | 43·0 (34·9–52·4) | 3·8 (2·9–5·0) | < 0·001 |
| Other MM combinations | 44 | 9 245·3 | 4·8 (3·5–6·4) | 0·9 (0·6–1·3) | 0·528 |
| **Non–AIDS-related mortality** |  |  |  |  |  |
| **Number of conditions** |  |  |  |  |  |
| 0 | 138 | 27 446·4 | 5·0 (4·2–5·9) | Reference | – |
| 1 | 941 | 129 169·0 | 7·3 (6·8–7·8) | 1·6 (1·3–2·0) | < 0·001 |
| ≥2 | 372 | 30 664·1 | 12·1 (10·9–13·4) | 2·0 (1·6–2·5) | < 0·001 |
| **Combinations** |  |  |  |  |  |
| No comorbidity | 138 | 27 446·4 | 5·0 (4·2–5·9) | Reference | – |
| Single condition | 941 | 129 169·0 | 7·3 (6·8–7·8) | 1·6 (1·3–2·0) | < 0·001 |
| Hypertension–diabetes–dyslipidaemia doublet | 267 | 19 116·2 | 14·0 (12·3–15·8) | 2·1 (1·6–2·6) | < 0·001 |
| Hypertension–diabetes–dyslipidaemia triad | 76 | 2 302·6 | 33·0 (26·0–41·3) | 3·9 (2·8–5·4) | < 0·001 |
| Other MM combinations | 29 | 9 245·3 | 3·1 (2·1–4·5) | 0·8 (0·5–1·3) | 0·407 |

MM, metabolic multimorbidity; PWH, people with HIV; PY, person-years; hypertension–diabetes–dyslipidaemia doublet, any two of hypertension, diabetes, and dyslipidaemia; hypertension–diabetes–dyslipidaemia triad, coexistence of hypertension, diabetes, and dyslipidaemia. Mortality rates (per 1000 person-years) and adjusted hazard ratios (aHRs) with 95% confidence intervals (CIs) were estimated using separate time-dependent Cox proportional hazards models. One model used metabolic comorbidity counts (0, 1, and ≥2 conditions), and the other used MM combinations (no comorbidity, single condition, hypertension–diabetes–dyslipidaemia doublet, hypertension–diabetes–dyslipidaemia triad, and other MM combinations) as the primary exposure, with the no comorbidity group as the reference, while adjusting for covariates.

***Supplementary Table 20. Relationship between numbers and combinations of metabolic conditions, modelled as time-varying exposure with a 180-day lag time after MM onset, and all-cause mortality***

| **Group of metabolic comorbidity** | **Mortality** | **PY** | **Mortality rate per 1000 PY (95% CI)** | **Adjusted HR (95% CI)** | ***p* value** |
| --- | --- | --- | --- | --- | --- |
| **Number of conditions** |  |  |  |  |  |
| 0 | 197 | 27 446·4 | 7·2 (6·2–8·3) | Reference | – |
| 1 | 1 327 | 129 169·0 | 10·3 (9·7–10·8) | 1·7 (1·5–2·0) | < 0·001 |
| ≥2 | 455 | 30 664·1 | 14·8 (13·5–16·3) | 1·9 (1·6–2·3) | < 0·001 |
| **Combinations** |  |  |  |  |  |
| No comorbidity | 197 | 27 446·4 | 7·2 (6·2–8·3) | Reference | – |
| Single condition | 1 327 | 129 169·0 | 10·3 (9·7–10·8) | 1·7 (1·5–2·0) | < 0·001 |
| Hypertension–diabetes–dyslipidaemia doublet | 322 | 19 116·2 | 16·8 (15·1–18·8) | 2·0 (1·6–2·4) | < 0·001 |
| Hypertension–diabetes–dyslipidaemia triad | 94 | 2 302·6 | 40·8 (33·0–50·0) | 3·9 (3·0–5·0) | < 0·001 |
| Other MM combinations | 39 | 9 245·3 | 4·2 (3·0–5·8) | 0·9 (0·6–1·2) | 0·437 |

MM, metabolic multimorbidity; PY, person-years; HR, hazard ratio; CI, confidence interval; hypertension–diabetes–dyslipidaemia doublet, any two of hypertension, diabetes, and dyslipidaemia; hypertension–diabetes–dyslipidaemia triad, coexistence of hypertension, diabetes, and dyslipidaemia. This sensitivity analysis extended the lag period for attributing mortality to metabolic multimorbidity from 90 days (main analysis) to 180 days. Cox models were adjusted for the same covariates as primary analyses.

***Supplementary Figure 1. Radar plots of metabolic condition burden among participants aged ≤45 years***


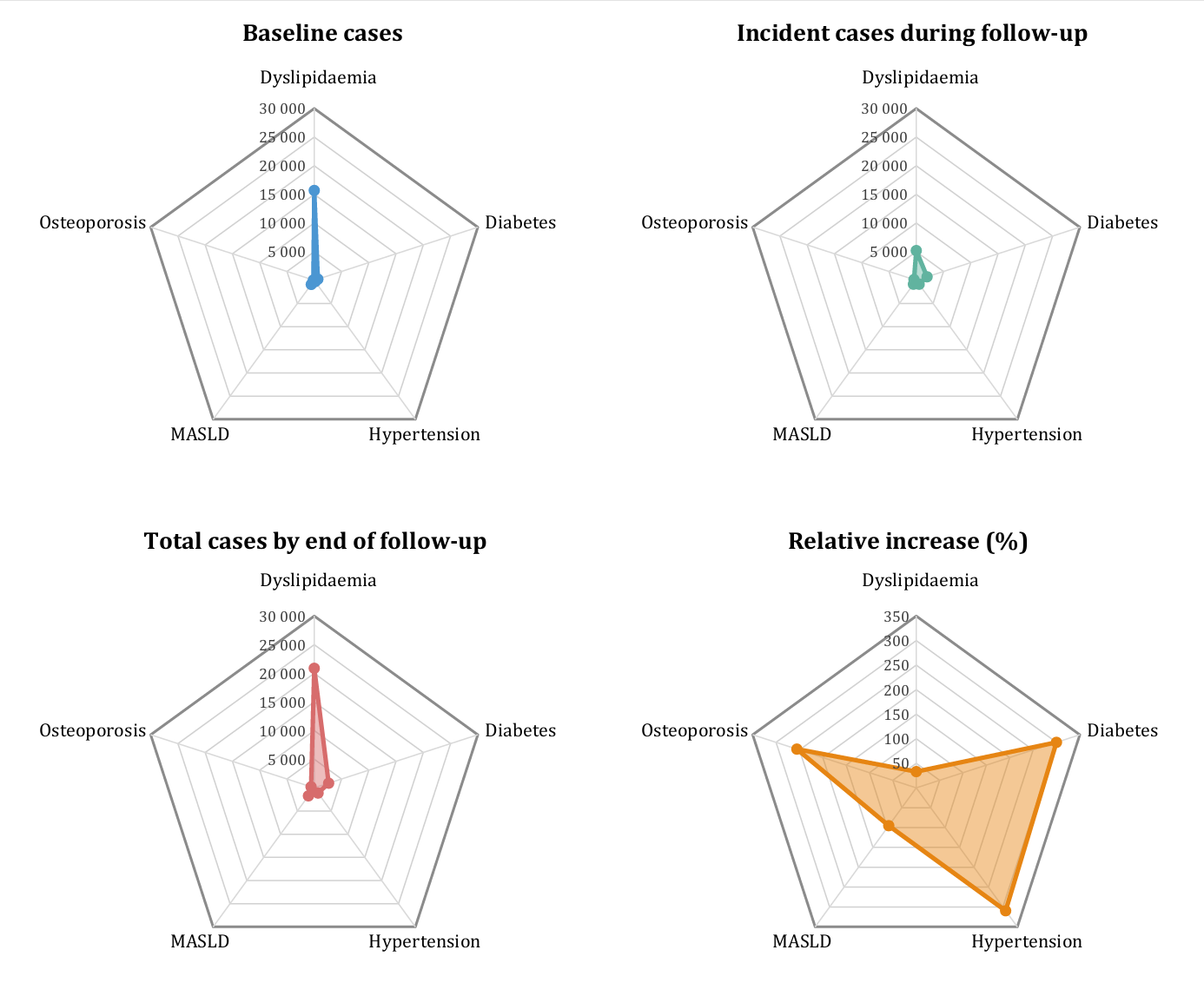
Radar plots show baseline cases, incident cases during follow-up, and total cases by end of follow-up, and relative increase for each metabolic condition. PWH: people with HIV; MASLD: metabolic dysfunction–associated steatotic liver disease.


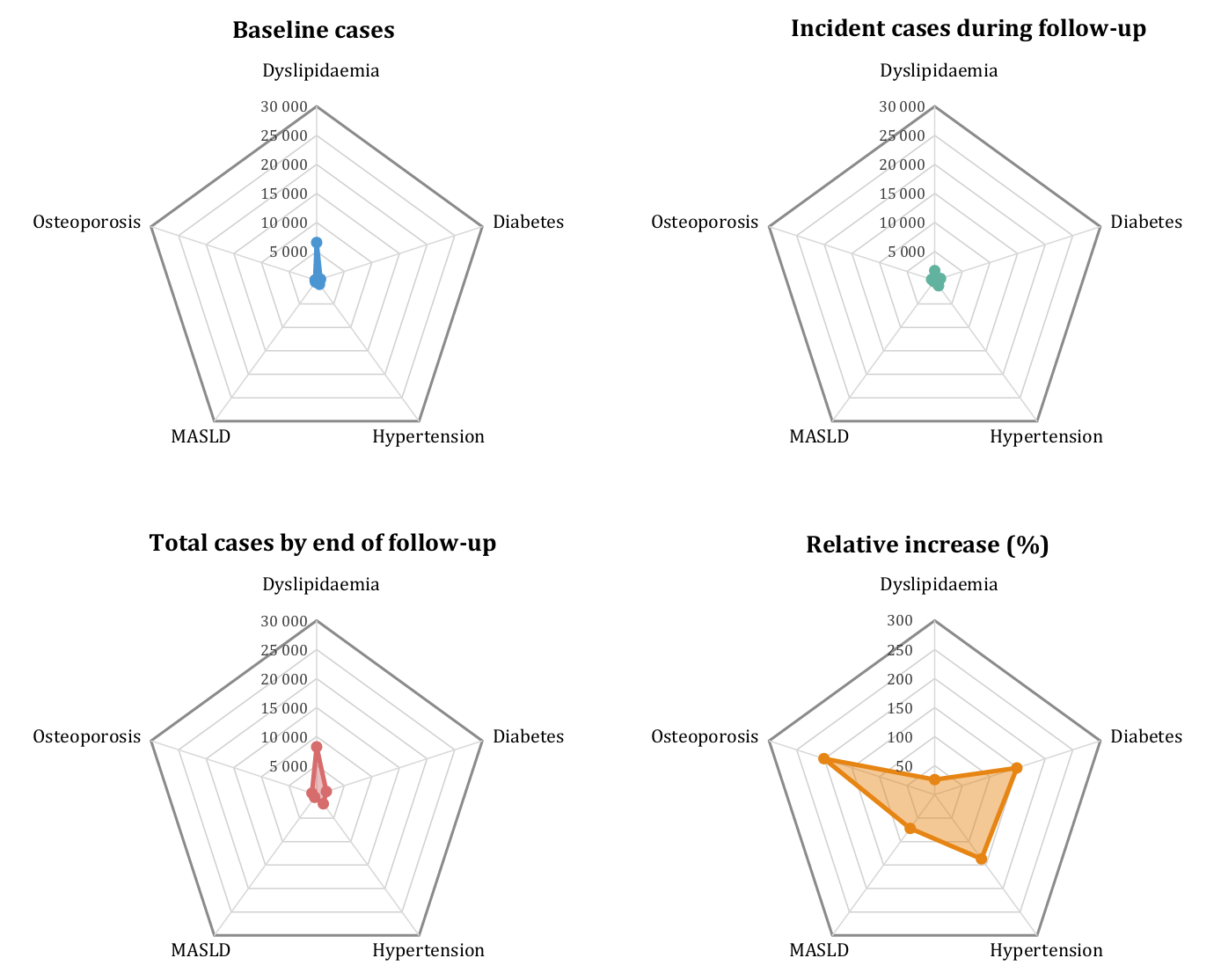
***Supplementary Figure 2. Radar plots of metabolic condition burden among participants aged >45 years***

Radar plots show baseline cases, incident cases during follow-up, and total cases by end of follow-up, and relative increase for each metabolic condition. PWH: people with HIV; MASLD: metabolic dysfunction–associated steatotic liver disease.

***Supplementary Figure 3. Exploratory clustering of metabolic multimorbidity patterns at 1, 3, and 5 years after ART initiation***


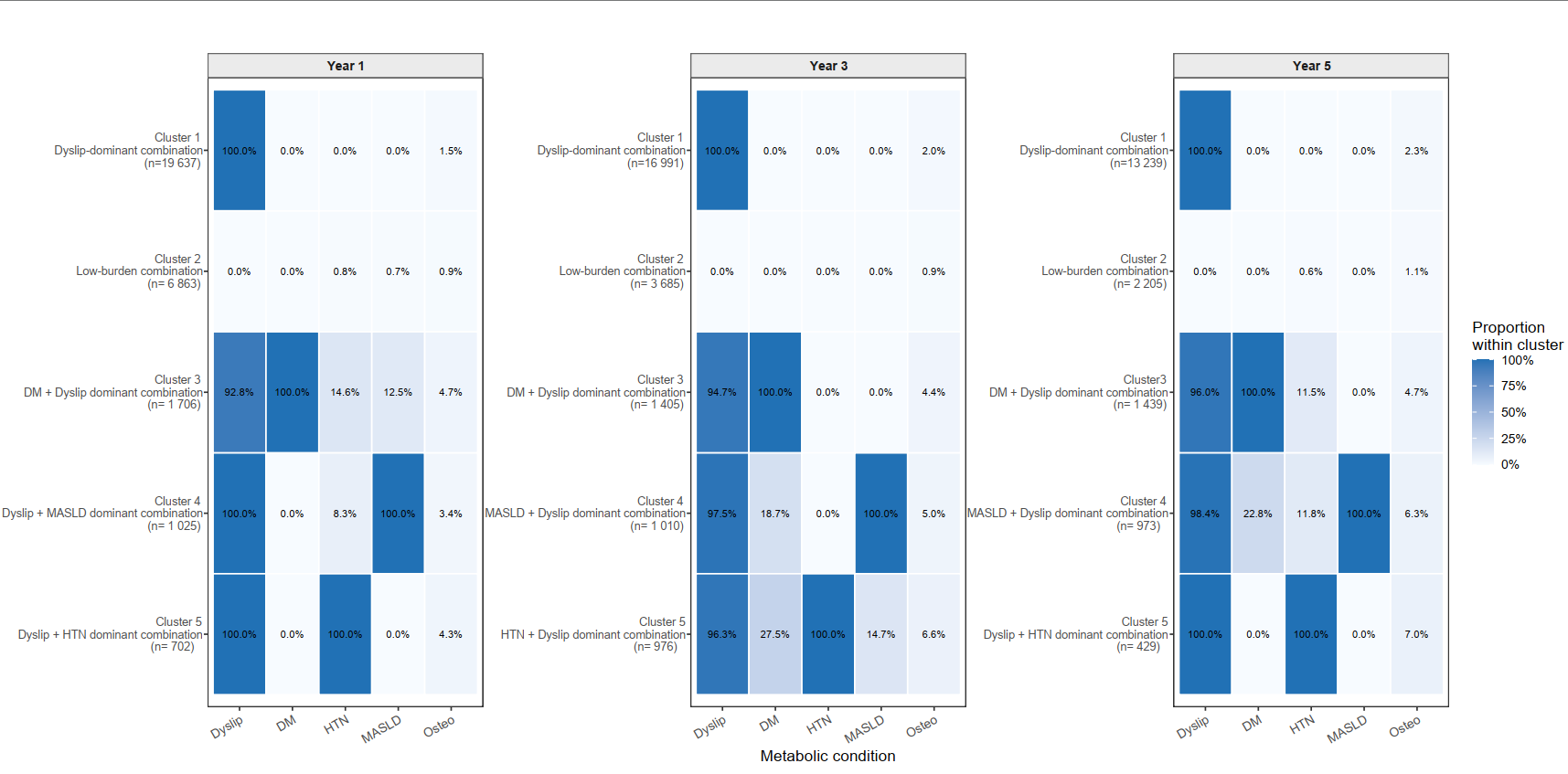


Heatmaps show the within-cluster proportion of participants with each metabolic condition at the 1-, 3-, and 5-year landmarks after ART initiation. Clusters were identified using landmark-based partitioning around medoids (PAM) clustering with Gower distance, based on binary indicators of cumulative metabolic conditions. Numbers in parentheses denote cluster size. ART, antiretroviral therapy; Dyslip, dyslipidaemia; DM, diabetes mellitus; HTN, hypertension; MASLD, metabolic dysfunction-associated steatotic liver disease; Osteo, osteoporosis.

***Supplementary Figure 4. Metabolic-related mortality rates by baseline MM and participant characteristics***


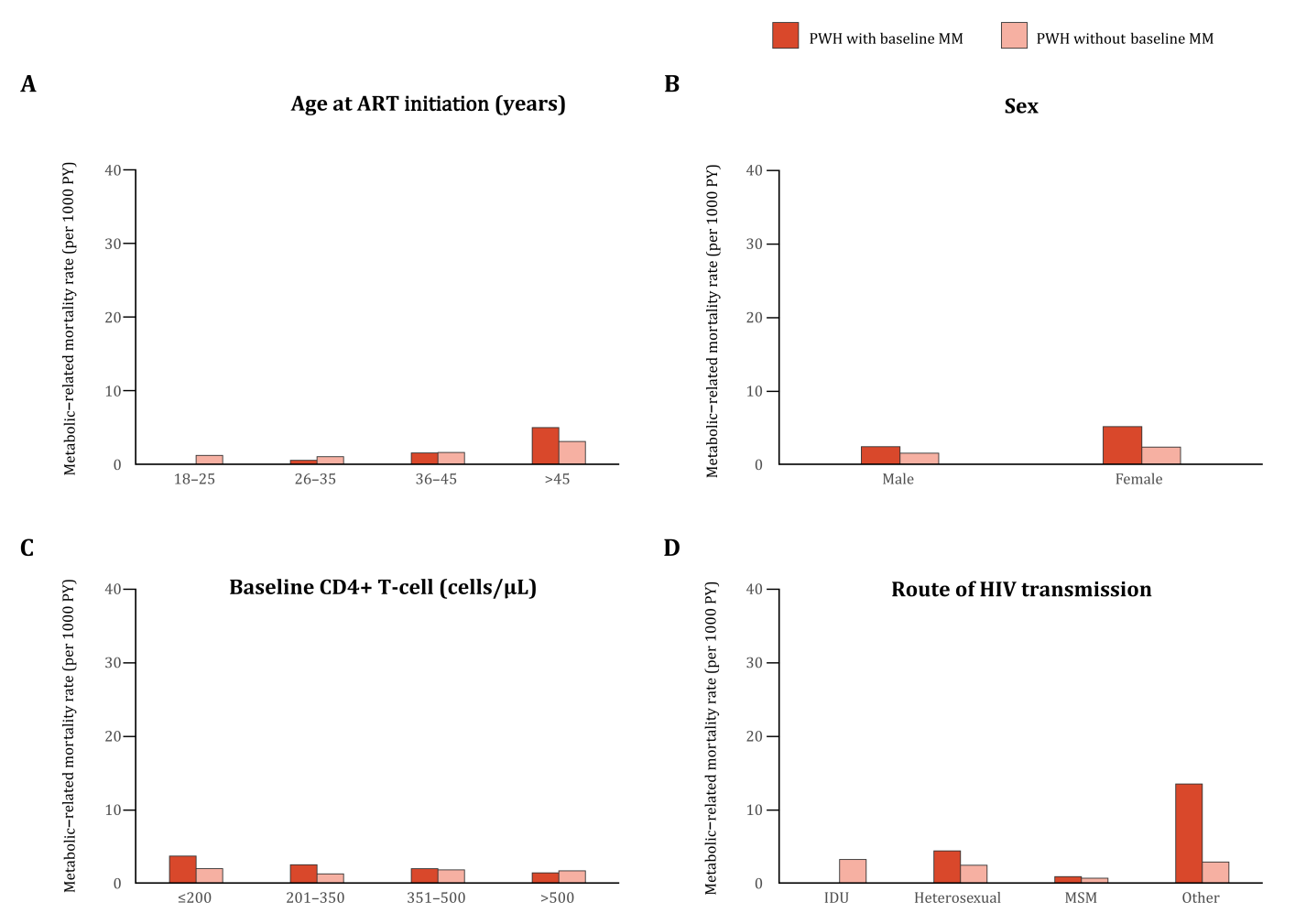
Bars show metabolic-related mortality rates (per 1000 PY) for participants with baseline MM versus those without baseline MM. Panel (A) shows the rates by age at ART initiation. Panel (B) compares the rates between male and female participants. Panel (C) shows the rates by baseline CD4+ T-cell count categories. Panel (D) displays the rates based on the route of HIV transmission. The first bar represents participants with baseline MM, and the second bar represents participants without baseline MM.

MM: metabolic multimorbidity; PY: person-years; ART, antiretroviral therapy; IDU: injection drug use; MSM: men who have sex with men.


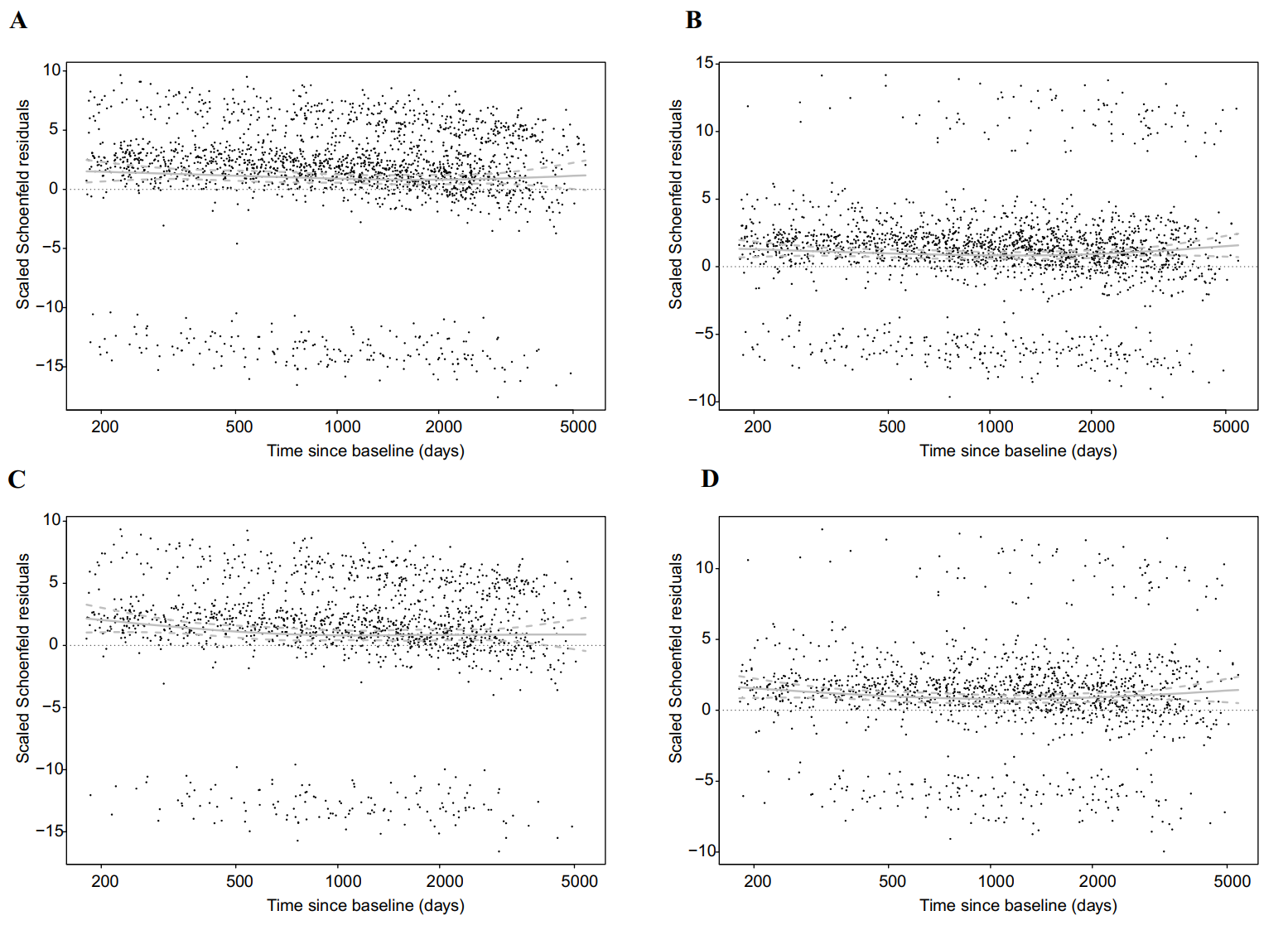
***Supplementary Figure 5. Scaled Schoenfeld residuals for assessing proportional hazards assumption in Cox models***

1. Number of conditions (all-cause mortality) (B) Number of conditions (non–AIDS-related mortality) (C) Combinations (all-cause mortality)
2. Combinations (non–AIDS-related mortality)

This figure displays the scaled Schoenfeld residuals plotted against time since baseline (in days) for different variables in Cox proportional hazards models. The smoothed grey lines indicate trends in the scaled Schoenfeld residuals over time, suggesting departures from the proportional hazards assumption.
